# Dual-Wavelength Simultaneous Patterning of Degradable Thermoset Supports for One-Pot Embedded 3D Printing

**DOI:** 10.1021/acscentsci.5c00337

**Published:** 2025-06-04

**Authors:** Isabel Arias Ponce, Bryan Moran, Craig J. Hawker, Maxim Shusteff, Sijia Huang

**Affiliations:** † Materials Department, 8786 University of California Santa Barbara, Santa Barbara, California 93106, United States; ‡ Materials Engineering Division, 4578 Lawrence Livermore National Laboratory, Livermore, California 94550, United States; § Department of Chemistry & Biochemistry, University of California, Santa Barbara, California 93106, United States; ∥ Materials Research Laboratory, 8786 University of California Santa Barbara, Santa Barbara, California 93016, United States; ⊥ Department of Chemical Engineering, University of Utah, Salt Lake City, Utah 84112, United States; # Department of Materials Science & Engineering, University of Utah, Salt Lake City, Utah 84112, United States

## Abstract

Vat photopolymerization (VP) techniques have enabled the fabrication of complex geometries while balancing high precision and fast processing times. 3D printed objects are traditionally built layer-by-layer with newly cured layers being structurally supported by previous ones. Fabricating unsupported features such as overhangs and arches risks misalignment and sagging, limiting the range of accessible designs. To overcome this issue, support structures are fabricated along with the primary object as temporary scaffolds that provide stability and conserve print fidelity. For VP specifically, patterning dissolvable sacrificial supports is attractive to avoid manual removal after printing. In this study, we demonstrate a base-degradable thermoset to pattern print supports in a one-pot formulation along with the primary structural material. Efficient printing is enabled using a dual-wavelength negative imaging (DWNI) DLP printer that patterns the degradable thermoset with visible light and the permanent network with UV light, which are simultaneously projected using a single digital micromirror device (DMD). Printed objects undergo thermal postprocessing to enhance the final conversion of the primary material, after which thermoset supports are degraded in a basic, aqueous solution. This approach provides a robust method for the dual-wavelength patterning of sacrificial thermoset supports, broadening the range of accessible 3D printable materials and geometries.

## Graphical abstract

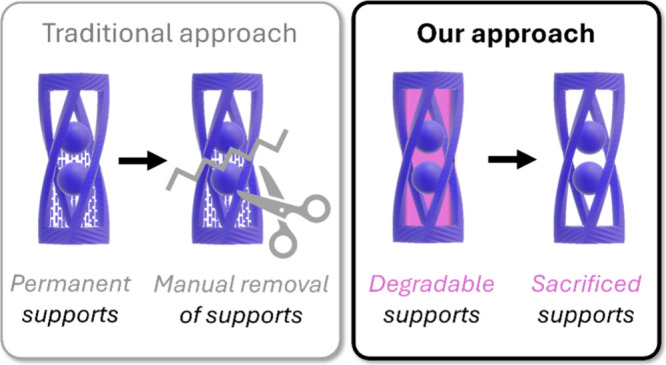

## 1 Introduction

In traditional layer-wise 3D printing, supports are frequently added to make certain geometries printable, in particular where layers are unconnected to previous ones, such as in spans, cantilevers, and overhangs.^1–3^ Supports are most often made of the same material which causes unnecessary material consumption and requires manual removal that is labor intensive and can leave surface defects. In powder-fusion approaches, such as selective laser sintering (SLS) of polymers or selective laser melting (SLM) of metals, the unfused powder bed acts as a support while lasers selectively fuse materials.^4,5^ In other classes of 3D printing, depositing a second, removable material to support the primary material is a common strategy.^6,7^ This includes commercial offerings, such as the Stratasys SR- family of mild-base soluble polymers for fused deposition modeling (FDM) and Solidscape Melt water-soluble wax for droplet jetting. Similarly, a class of printing approaches using gel, slurry or viscoelastic support baths for extrusion-based printing has emerged, sometimes termed freeform 3D printing.^8–11^ These embedded printing techniques remove geometric constraints, provide stability and are essential for the fabrication of pores, channels, and nonassembly articulated geometries with diverse applications including vascularized scaffolds for tissue engineering, fluidic structures, ultralight lattices for aerospace applications, and interlocking mechanisms such as joints or hinges.^12–14^

Vat photopolymerization (VP) methods, in which structures are produced by solidifying light-sensitive liquid resins, are attractive due to their ability to pattern objects with high resolution and fast processing times at low cost.^15–17^ In the most developed layer-based VP technologies, including stereolithography (SLA) and digital light processing (DLP), adding a secondary support material is difficult to implement because of the need to exchange resins and the required curing of distinct polymer networks. Previous investigations exploring sacrificial supports for VP have used a multimaterial resin-exchanging system, grayscale patterning, and a cooling device to pattern ice-based supports.^18–20^ However, these approaches require specialized hardware for material exchange and cooling as well as washing steps between resin exchanges to prevent copolymerization between dissolvable and permanent materials. Some VP approaches have been developed which allow support-free printing. For example, tomographic volumetric additive manufacturing (VAM) enables layer-less fabrication of complex objects by exposing resin to light projections from multiple angles.^21,22^ While it relies on a unique hardware configuration, custom optics, and advanced software algorithms to deliver controlled light dosages, it can build entire objects in a single, continuous exposure step, significantly enhancing throughput. A more well-developed support-free VP technology is two-photon or multiphoton printing (we refer to these collectively as 2PP), which enables support-free structure fabrication by confining polymerization to a submicron volume in which two or more photons are absorbed simultaneously.^23,24^ Despite their geometric versatility and high resolution, 2PP processes are typically low throughput and have limited build volumes, making them more suitable for high-precision and small-scale applications.

In this context, eliminating the need to add supports in layered VP technologies can be impactful primarily because SLA and DLP printing has gained commercial adoption across multiple industries. These technologies are more broadly accessible than VAM or 2PP, requiring fewer specialized optical components than VAM and allowing for larger build volumes than 2PP. Introducing dual-wavelength multimaterial resins to enable SLA or DLP printing of degradable supports reduces the barrier to fabricating complex, support-free objects. Leveraging orthogonal chemistries in a one-pot resin can enhance efficiency and dimensional accuracy by patterning permanent features and soluble supports based on wavelength. Page and colleagues described a hybrid epoxy-acrylate multimaterial resin for dual-wavelength 3D printing which enabled objects with spatial and wavelength-controlled polymerization.^25^ By carefully selecting the photoinitiating components, a radical polymerization was triggered with visible light and a hybrid epoxy-acrylate polymerization was triggered with UV light. Similarly, recent work by Diaco and colleagues used a dual-wavelength printer to fabricate multimaterial objects with dissolvable, thermoplastic supports.^26^ However, as thermoplastics typically require high concentrations to solidify, the multimaterial resin in this strategy contained a relatively high proportion of the dissolvable acrylate (60 wt %) used for the supports, reducing the available permanent epoxy material (40 wt %). This is undesirable for the final mechanical properties and structural integrity of the printed parts once supports are removed.

Taking inspiration from these approaches to generate spatially distinct polymer networks, we aimed to design a one-pot resin printable with multiple light wavelengths with a degradable component to be used as sacrificial support material. As a complementary approach to dissolvable thermoplastic supports, we have developed an aqueous, base-degradable thermoset material to maximize the primary epoxy content and enhance stability of the final parts. Furthermore, to take advantage of orthogonal chemistries, we use a custom dual-wavelength negative imaging (DWNI) DLP printer, in which both UV and visible light are simultaneously projected by a single digital micromirror device (DMD) which selects for either light beam based on the micromirror angle.^27^ Using a single DMD potentially enhances resolution as it eliminates the need for multiple DMD alignments. In addition, simultaneously projecting both light sources leads to a decrease in print time by up to 50% compared to printers which can only expose them sequentially, assuming the same layer time is allocated to both wavelengths. The degradable thermoset is patterned with visible light (405 nm) which triggers free-radical polymerization of a (meth)­acrylate network. The permanent epoxy network is patterned with UV light (365 nm) which triggers both radical and cationic polymerization. In addition, aqueous degradation conditions may potentially be more favorable for downstream biocompatibility of the resulting structure. This work, in the context of other investigations of multiwavelength orthogonal chemistries contributes to a palette of options for photopolymer resin printing for dissolvable supports. Overall, this combination of characteristics increases the versatility of VP systems for overcoming limitations in fabricating unsupported geometries by embedding permanent objects within degradable materials to enhance print fidelity.

## 2 Results and Discussion

### 2.1 Development of a One-Pot Dual-Wavelength Resin

To design an effective one-pot, dual-wavelength resin to pattern degradable supports, the resin must meet three key criteria: 1) fast reaction kinetics for epoxy and (meth)­acrylate polymerization under UV and visible light; 2) a thermoset (meth)­acrylate network that degrades under mild conditions; 3) photoinitiator wavelength selectivity between UV and visible light. We developed a one-pot, dual-wavelength resin with epoxy monomers and (meth)­acrylate monomers (Figure 1). The selected epoxy monomers include 3,4-epoxycyclohexylmethyl-3′,4’-epoxycyclo hexane carboxylate (ECC) as a cross-linker and 3-ethyl-3-oxetanemethanol (OXA) as a comonomer to enhance cationic polymerization kinetics.^25^ For the (meth)­acrylate monomers, we selected 4-acryloylmorpholine (ACMO) as a comonomer and methacrylated sebacic acid (MSA) as a cross-linker. ACMO can accelerate radical polymerization kinetics and has been widely used in photopolymer studies due to its high glass transition temperature (*T*_g_ ∼ 155 °C), ultralow viscosity (12 cP) and quick gelation to minimize oxygen inhibition.^28^ To introduce degradability, MSA was selected for its fast polymerization kinetics and hydrolytic degradation into nontoxic sebacic acid products.^29^ MSA was synthesized following a previously published procedure (SI S1.1).^30^ For the initiation system, the radical photoinitiator phenylbis­(2,4,6-trimethylbenzoyl) phosphine oxide (BAPO) efficiently absorbs both UV light (365 nm) and visible light (405 nm) to initiate free radical polymerization and allow for cross-linking of the degradable thermoset network (Figure S3). The photoacid generator diphenyl­[4-(phenylthio)­phenyl] sulfonium hexafluoro antimonate (DHS) only selectively absorbs UV light (365 nm) to initiate both radical and cationic polymerization, yielding a permanent network (Figure S3). Additionally, the photoabsorber Sudan I absorbs at both wavelengths to control cure depth during printing. Detailed mass and molar resin composition can be found in SI S2.1.

**Figure 1:**
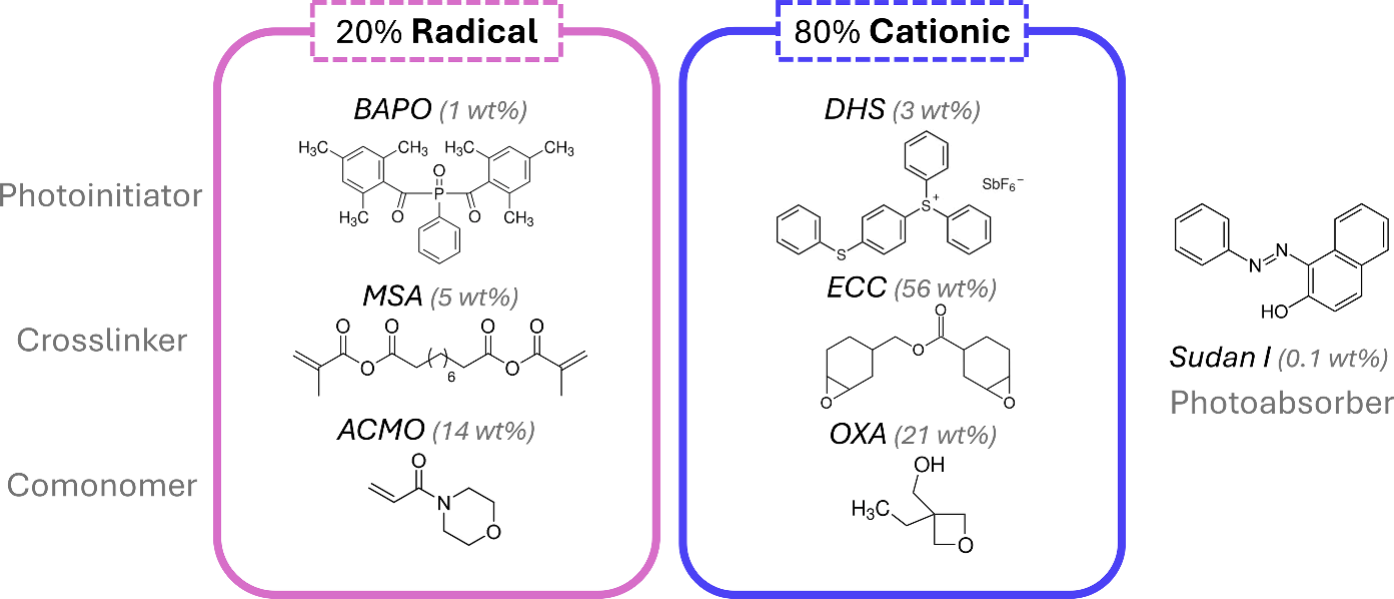
Dual-wavelength resin incorporates both radical and cationic networks in one pot, including photoinitiators, cross-linkers, and comonomers for each reaction.

Compared to previous dual wavelength resins designed for thermoplastic sacrificial supports, the key advantage of this work is the incorporation of a degradable anhydride-based (meth)­acrylate thermoset network, which maximizes the final cationic network concentration in the one pot system. The inclusion of MSA as a cross-linker significantly reduces the required loading of the radical monomers due to its thermoset nature which allows for solidification at lower concentrations than with thermoplastics. As a result, the radical network solidifies with only 20 wt % (meth)­acrylate composition, doubling the cationic network concentration of current one-pot systems to 80 wt %. The higher cationic content enhances the overall structural integrity of the final object due to its higher cross-link density and reduced free volume. In addition to maximizing the mechanical properties of the epoxy network, the (meth)­acrylate thermoset network shows a glass transition (Tg ∼ 67 °C, Figure S9) but no melting transitions, reducing the risk of shape deformation during heat postprocessing.

### 2.2 Probing Wavelength Selectivity of the Resin

The wavelength-selectivity of the cationic and radical polymerizations was assessed via transmission mode real time Fourier Transform Infrared Spectroscopy (FTIR)­with coupled LED lightguides at 365 and 405 nm wavelengths (Detailed methods in SI S2.3). To avoid peak convolution, the (meth)­acrylate and epoxy network resins were prepared and monitored separately at 20 and 80 wt % concentrations respectively as shown in Table S1. The residual concentration for both resins was completed with propylene carbonate as it has low volatility and dissolves solid components. Light was irradiated onto the resin samples after 30 s of initial data collection. Under visible light (405 nm, 30 mW/cm^2^), (meth)­acrylate monomers reached 50% conversion within ∼ 70 s of exposure, equivalent to 2100 mJ/cm^2^ of radiant exposure (Figure 2, top). In contrast, epoxy monomers did not reach significant conversion (<5%) even after ∼ 270 s of exposure under 405 nm light. Low conversion of the cationic network was expected due to minimal absorption of the photoacid generator in the visible light region, thereby limiting initiation of the cationic polymerization (Figure S3). The selectivity of visible light to initiate radical but not cationic polymerizations allows 405 nm irradiation to be used to fabricate the degradable supports.

**Figure 2:**
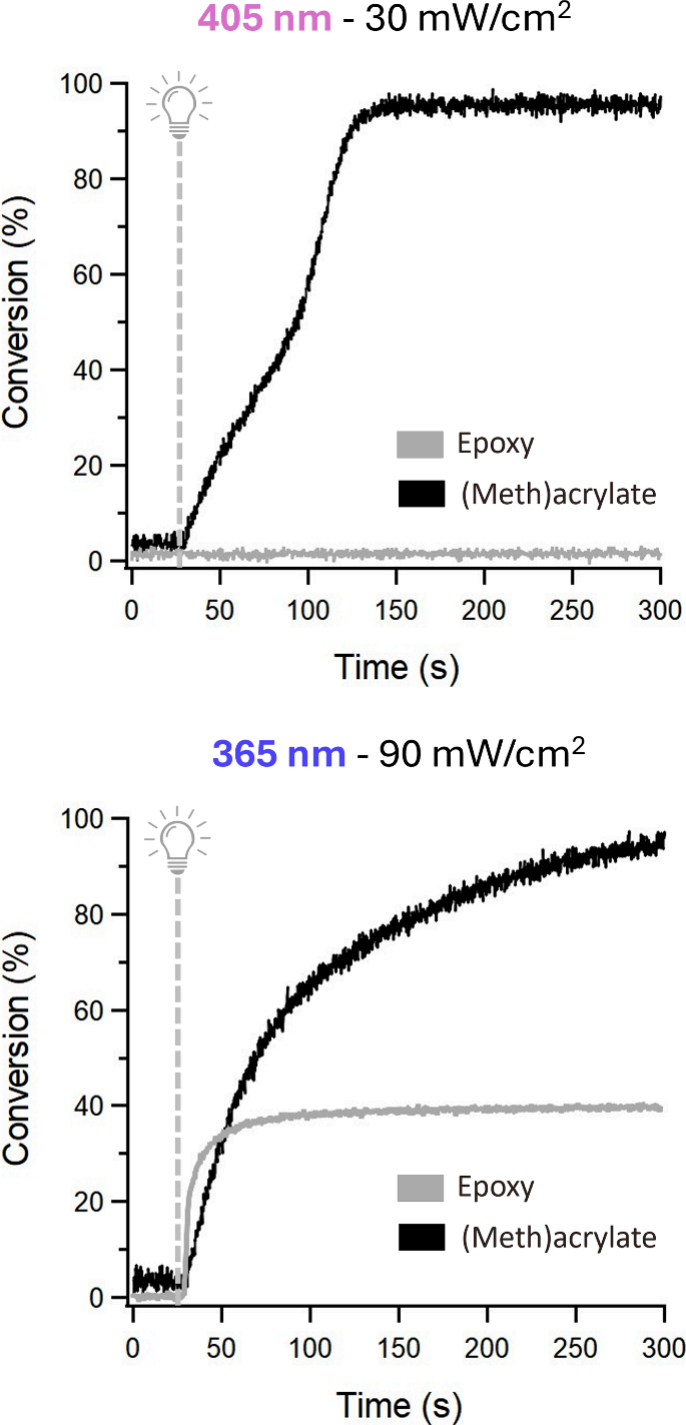
Transmission FT-IR was used to track the conversion of both (meth)­acrylate and epoxy functional groups under 405 and 365 nm light. 405 nm light triggered only the radical reaction while 365 nm light triggered both the radical and cationic reactions.

Under UV light (365 nm, 90 mW/cm^2^), epoxy monomers reached approximately 40% maximum conversion within ∼ 70 s of exposure, equivalent to 6300 mJ/cm^2^ of radiant exposure (Figure 2, bottom). Heat postprocessing of the epoxy materials increased the cationic conversion up to 57% (Figure S4). (Meth)­acrylate monomers under UV light initially showed faster polymerization kinetics than under visible light. However, the rate decreased after 100 s of irradiance and was ultimately surpassed by visible light polymerization. The initial fast rate of UV triggered radical polymerization may be due to higher photoinitiator absorption presumably leading to faster initiation (Figure S3). The high concentration of free radicals as well as excessive recombination and termination events could be responsible for the subsequent polymerization slowdown. Photorheology studies confirmed kinetic results via gelation measurements (SI S2.4).

While thermal treatment improved thermomechanical properties, it may also compromise resolution due to monomer diffusion between degradable and nondegradable regions which may result in epoxy conversion in the visible light cured, degradable regions. Therefore, introducing photosensitizers with high absorptivity in the UV region (365 nm), such as 3,6-dimethoxy-9H-thioxanthen-9-one (MeOTX), into the resin composition could enhance the efficiency of photoacid generators and potentially reduce the need for thermal postcuring, as recently demonstrated by Mason and collaborators.^31^ In addition, varying the OXA/ECC monomer concentration could help enhance epoxy conversion rates, allowing for shorter layer times, and increasing print efficiency.

### 2.3 Dual Wavelength Negative Imaging (DWNI) DLP Printer

The dual-wavelength negative imaging (DWNI) DLP printer is a custom-made system and can independently control mirrors in the digital micromirror device (DMD) to irradiate either visible light (λ_1, 405 nm_) or UV light (λ_2, 365 nm_) based on their tilt position. Due to the nature of the optical arrangement, pixels in the projected image are negatives of each other and irradiate one of two wavelengths, giving the “negative imaging” and “dual wavelength” properties of the DWNI stereolithography system (Figure 3B).

**Figure 3:**
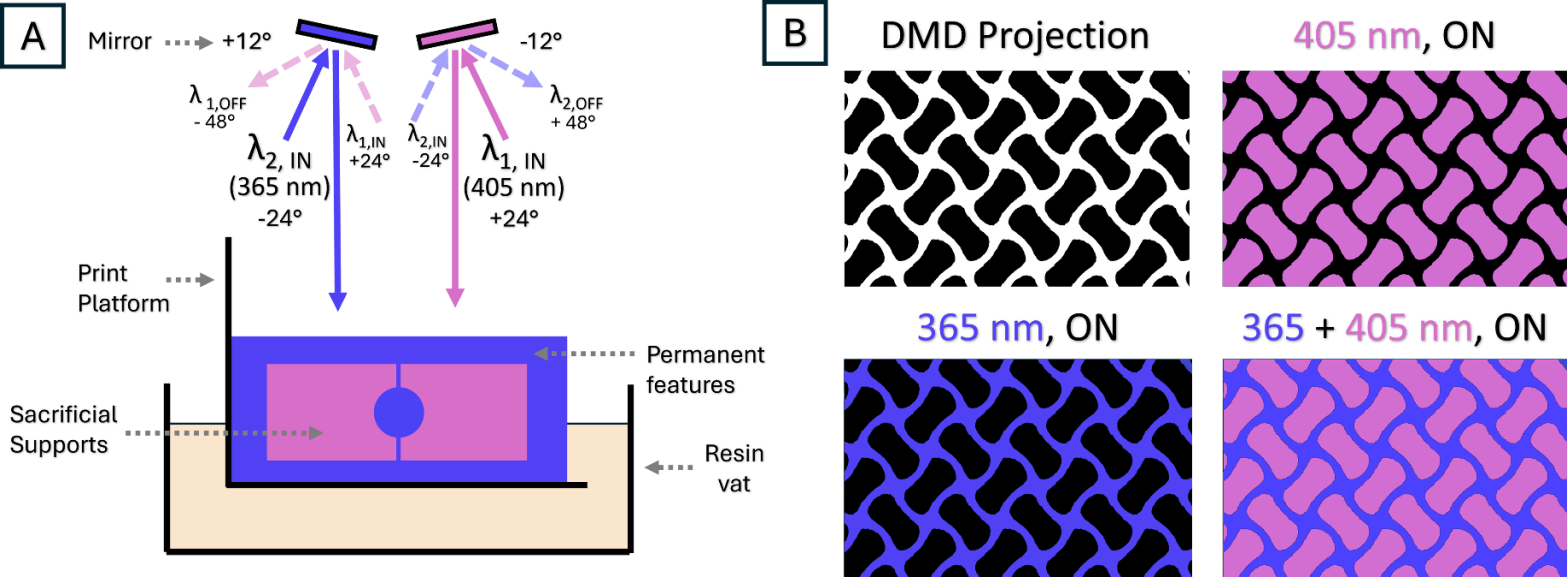
(A) The dual-wavelength negative imaging (DWNI) DLP printer can independently control mirrors in the digital micromirror device (DMD). By toggling the mirror angles, different wavelengths of light can be projected to different pixels in the same layer. (B) 2D black/white images are uploaded to the DMD to pattern each layer. Black areas are filled with 405 nm light and white areas are filled with 365 nm light. Each wavelength can be projected both independently and simultaneously.

When micromirrors are positioned to the −12° orientation, beamline 1 (λ_1, 405 nm_) which is aligned at +24° degrees reflects off the mirrors and exits from the DMD face at 0° (surface normal), as shown in Figure 3A. The reflected light (λ_1, 405 nm_) is then collected by system optics and projected as images on the working surface. However, toggling the micromirrors to the +12° position changes the direction of the light to −48° which falls outside the aperture of the collection optics no longer reaching the working surface. This is the essence of all DMD operations in microstereolithography which produce images by toggling micromirrors between −12°/+12° position turning pixels ON/OFF.

In the DWNI system, a second light source beamline 2 (λ_2, 365 nm_) is aligned as a mirror image of beamline 1 at −24° degrees, reflects off mirrors in the +12° position, and reaches the working surface at 0° (surface normal), as depicted in Figure 3A. Therefore, micromirror toggling produces opposite ON/OFF results between the two beamlines and both of the micromirror positions (−12°/+12°) reflect light toward the surface normal, to the collection optics, and onto the working surface, only changing the ON wavelength (λ_1, 405 nm_ or λ_2, 365 nm_) for each pixel. As only one DMD is projected onto the working surface, issues with overlapping, magnification matching, rotation, position focusing, need for dichroic beam splitters and polarization optics, and cost from a second DMD are eliminated. Finally, there is virtually no leakage of one wavelength into another due to the nature of the DMD strongly rejecting the OFF wavelength. Since both wavelengths are projected simultaneously, print time is reduced by up to 50% compared to dual-wavelength systems with a single DMD that expose sequentially, specifically in cases where the same layer time is allocated to each wavelength. A more detailed description of the 3D printer set up can be found in .

### 2.4 Resin Printability Optimization

To efficiently optimize resin printability and postprocessing parameters prior to 3D printing, working curves at varying radiant exposures were created for each wavelength. The one-pot resin was exposed in small square patterns, each with incremental exposure times (5–70 s) under either visible (405 nm, 30 mW/cm^2^) or UV light (365 nm, 90 mW/cm^2^). All square features were washed in organic solvent (isopropanol-acetone mixtures), thermally postprocessed at 110 °C for 15 min, and degraded in 5 M NaOH solution for 15 min at room temperature. The square features patterned with visible light (405 nm) survived solvent washes and thermal postprocessing but completely degraded after the base wash (Figure 4A, bottom). This result aligns with FTIR characterization, confirming that only (meth)­acrylate monomers undergo polymerization under visible light irradiation. Therefore, visible light printed features mainly consisted of the (meth)­acrylate-based degradable network. The anhydride groups in the (meth)­acrylate network undergo base-catalyzed hydrolysis and degrade into nontoxic compounds, making it a key aspect of green chemistry.^29,30^ Working curve plots of visible light irradiated features show complete loss of features following base degradation (Figure 4B, right).

**Figure 4:**
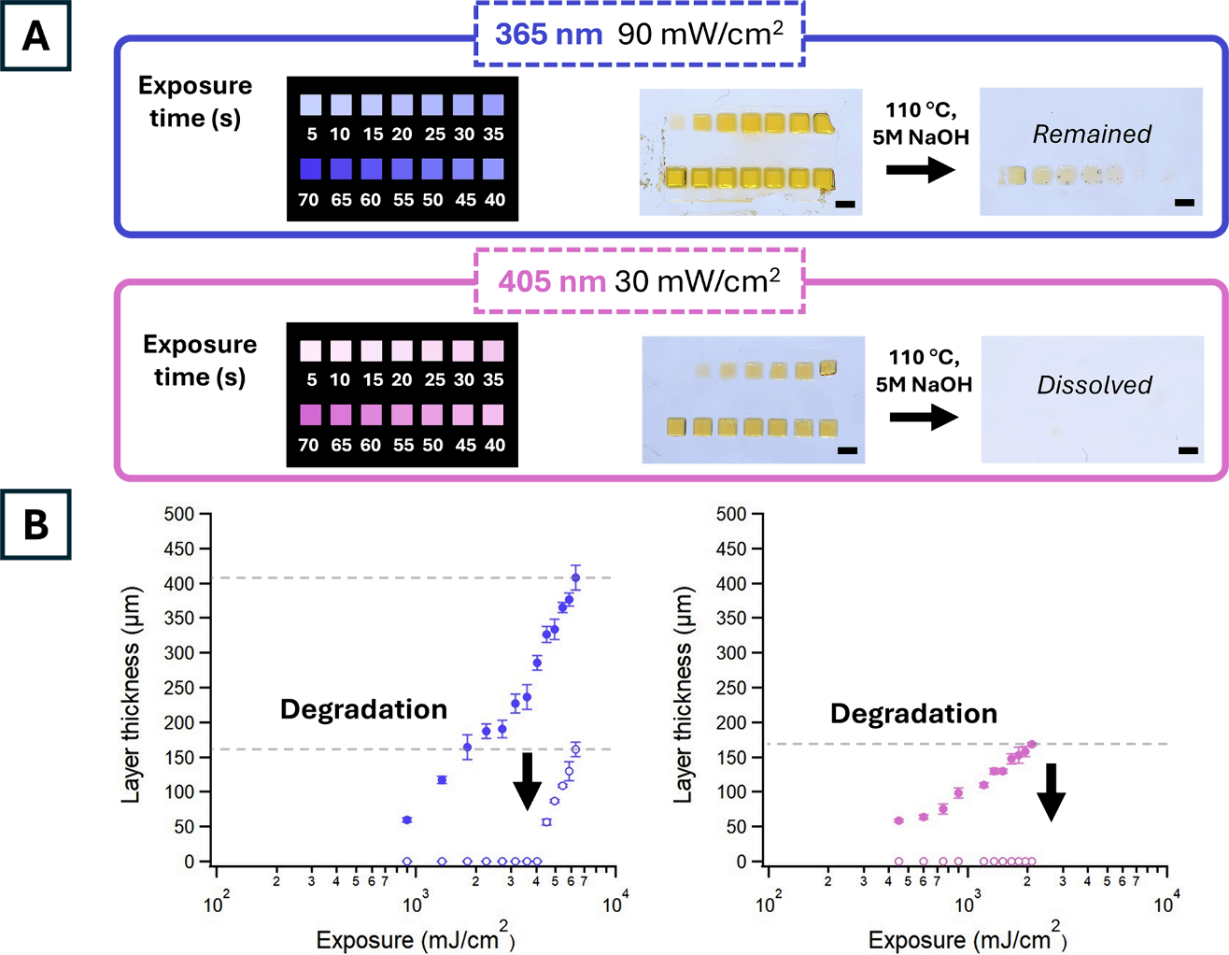
(A) Square features were patterned onto glass slides with varying exposure energies (mJ/cm^2^) for each wavelength (405 or 365 nm) by varying exposure times. Features irradiated with 405 nm light dissolved postdegradation in 5 M NaOH, while those irradiated with 365 nm light at the highest exposure energies survived. (B) 365 nm cured features with highest light exposure decreased in layer thickness postdegradation. Scale bars are 1 mm.

For the UV light patterned regions (365 nm), features patterned with high exposure energy (>4500 mJ/cm^2^) survived base degradation (Figure 4A, top), indicating that UV light printed features are mainly composed of epoxy networks. However, only the high UV irradiated features form an interconnected epoxy gel that resists hydrolytic degradation. Working curve plots of layer thickness (μm), also called cure depth (C_d_), vs exposure energy (mJ/cm^2^) of UV irradiated features show a decrease in thickness after the base degradation step (Figure 4B, left). Regions farthest from the light source and deeper into the resin are subject to higher resin absorption and scattering events which attenuate light. This results in lower UV exposures and insufficient cationic polymerization. These regions therefore have overall lower epoxy conversions and are more susceptible to degradation with base. The highest exposure times (∼70s) and exposure energies (405 nm 2100 mJ/cm^2^, 365 nm 6300 mJ/cm^2^) for both wavelengths were used for subsequent experiments as they patterned cure depths around 150 μm, ideal for printing with 50 μm layer thicknesses. It is worth noting that the exposure time needed to pattern nondegradable epoxy features (∼70s) could be longer than the time needed to fabricate a green body for postcuring using traditional supports. However, prolonged thermal postcuring of a green body could also allow monomers to migrate between degradable and nondegradable regions, reducing resolution and compromising the degradability of supports. In addition, manually removing traditional supports may compromise the integrity of the final parts made of stiff and brittle epoxy networks as in this study.

### 2.5 2D and 3D Printing Degradable Supports

After identifying optimal exposure energies (405 nm 2100 mJ/cm^2^; 365 nm 6300 mJ/cm^2^) and layer thickness (50 μm) to cure degradable and permanent regions, we next patterned 2D projections by irradiating both wavelengths simultaneously for 70s (Figure 5A). This enables print times to be reduced by ∼ 50% compared to commercial dual-wavelength systems that only irradiate one wavelength at a time, assuming equal layer times for both wavelengths. Checkerboard and cross patterned projections were fabricated onto glass slides and subsequently undergo solvent washes, thermal postprocessing, and base degradation. Regions patterned with visible light were efficiently removed while the UV irradiated regions survived processing steps as expected (Figure 5A). The degradation time of visible light cured materials was geometry and solvent dependent, with 2 mm thick discs degrading within 2 h in 5 M NaOH solution (Figure S7) and within 18 h in distilled water (Figure S8). We then printed taller objects with free-floating structures embedded within degradable supports (Figure 5B). Five interlocked rings, a ball-in-a-cage (Supplementary Video), and a helix with two balls structures were fabricated with our DWNI DLP embedded approach to prevent free-floating structures from moving during the print process. Following postprocessing steps, final parts were easily released from the degradable network, demonstrating the versatility and resolution of our approach.

**Figure 5:**
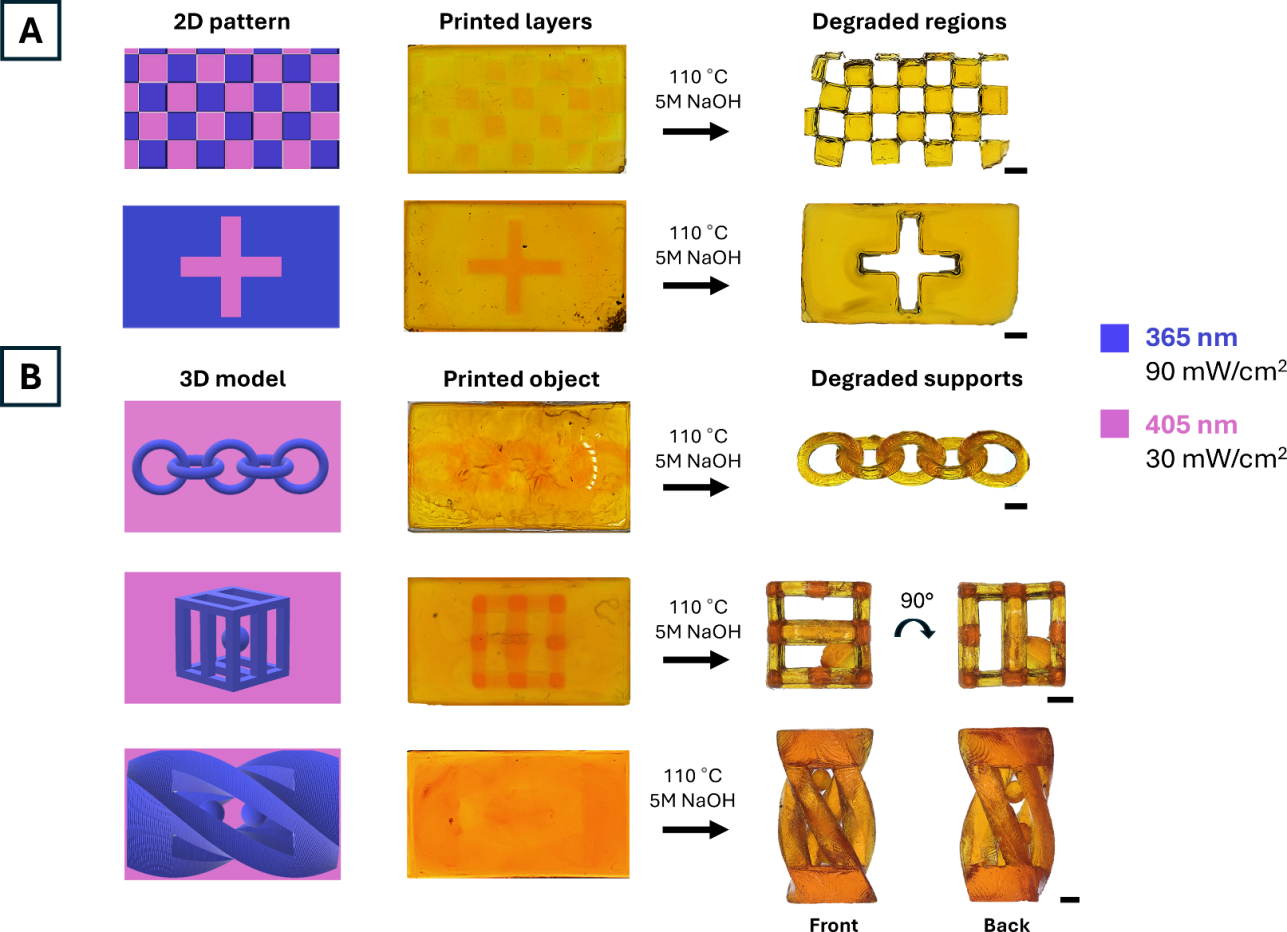
(A) Checkerboard and cross-pattern 10-layered projections and (B) 5-interlocked rings, ball-in-a cage and a helix with two balls structures were irradiated with 405 and 365 nm wavelengths to pattern sacrificial and permanent features. Permanent features are colored blue and degradable features are colored pink. Sacrificial features are degraded while permanent features are revealed after postprocessing. Scale bars are 1 mm.

Effects of the base degradation step on the surface roughness, cross-linking density/modulus, and print resolution of the final parts were also investigated. The surface roughness profile along a single layer revealed a homogeneous surface before (Figure S11A) and postdegradation (Figure S11C), while the surface roughness profile across multiple layers showed higher variability after base degradation (Figure S11D). This may be due to the base treatment removal of lower epoxy conversion regions between layers revealing discontinuities along the *z*-axis which could be addressed in the future by reoptimizing print parameters. In addition, dynamic mechanical analysis (DMA) of 365 nm cured parts showed that despite base degradation and a 35% reduction in cross-link density, the overall modulus remains governed by the robust epoxy network with a final rubbery modulus of 2.69 × 10^7^ Pa at 200 °C (Figure S12). Finally, resolution studies showed that the DWNI printing approach enhances shape retention and dimensional accuracy of final objects compared to using a single wavelength (Figure S13). This resolution enhancement might be due to the simultaneous formation of the degradable network in the negative or void regions, which temporarily constrains the epoxy network and limits its non-specific curing.

These objects would be challenging to fabricate with layer-based VP printing methods as they are not structurally supported by previous layers which demonstrates the advantage of the embedded DWNI DLP printing approach. In addition to enabling higher geometric versatility with its degradable supports, the DWNI printing method also fabricates objects with robust mechanical properties and enhanced resolution, making it a powerful strategy to widen the applications of DLP systems.

## 3 Conclusion

This study demonstrates a dual-wavelength, one-pot resin system to fabricate degradable anhydride-based thermoset supports and permanent epoxy regions to improve print fidelity in freeform additive manufactured parts. Complementary to previously reported thermoplastic dissolvable supports, the anhydride-based thermoset degradable supports in this study shows thermal stability during postprocessing while maintaining degradability. In addition, the degradable thermoset network solidifies at low concentrations, thereby maximizing the loading of the primary epoxy network and enhancing the structural integrity of the final parts. The one-pot resin was successfully patterned into degradable and permanent regions using a custom-made dual-wavelength negative imaging (DWNI) DLP printer. The DWNI DLP was configured to receive both wavelengths onto a single DMD and project them based the micromirror position, overcoming the need for multiple DMD alignments, reducing costs, and potentially cutting print time by 50% compared to commercial printers which expose multiple wavelengths sequentially. Printed parts were thermally treated to maximize conversion of the final network while maintaining degradability of the thermoset supports. In addition, the aqueous base degradation of the anhydride (meth)­acrylate network enables sustainable and damage-free support removal while preserving mechanical properties and enhancing the resolution of final parts. Our dual wavelength DLP printing strategy enables the facile fabrication of disconnected or unsupported geometries that would be difficult to achieve with layer-based manufacturing methods.

## Supplementary Material







## Data Availability

Dryad data sets are available free of charge at 10.5061/dryad.34tmpg4vq.

## Notes

Ball-in-a-cage structure (AVI)
Experimental details, figures, and tables, including synthetic protocols, resin formulations, UV–vis data, FTIR data, photorheology data, 3D printer setup, degradation data, and thermal characterization data (PDF)
Transparent Peer Review report available (PDF)
