# Supplementary material for: Dual-Wavelength Simultaneous Patterning of Degradable Thermoset Supports for One-Pot Embedded 3D Printing
Source: ACS Cent Sci. 2025 Jun 4;11(6):967–74. doi: 10.1021/acscentsci.5c00337 (PMC12203427; doi:10.1021/acscentsci.5c00337)
Supplement: Supplementary file 2 [file oc5c00337_si_002.pdf]

Supporting Information

Dual-wavelength simultaneous patterning of degradable thermoset supports for one-pot embedded 3D printing

Isabel Arias Ponce[1,2], Bryan Moran[2], Craig J. Hawker[1,3,4], Maxim Shusteff[2], Sijia Huang[2,5,6*]*

1. Materials Department, University of California Santa Barbara, Santa Barbara, California 93106, United States
2. Materials Engineering Division, Lawrence Livermore National Laboratory, Livermore, California, 94550, United States
3. Department of Chemistry & Biochemistry, University of California, Santa Barbara, California 93106, United States
4. Materials Research Laboratory, University of California Santa Barbara, Santa Barbara, California 93016, United States
5. Department of Chemical Engineering, University of Utah, Salt Lake City, Utah 84112, United States
6. Department of Materials Science & Engineering, University of Utah, Salt Lake City, Utah 84112, United States

*To whom correspondence should be addressed:

shusteff1@llnl.gov, sijia.huang@chemeng.utah.edu.

Supporting information

1. Materials……………………………………………………………………………S1
   1.1 Methacrylated sebacic acid (MSA) synthesis………………………………………S1
2. Dual-wavelength resin characterization………………………………………………S3
   2.1 Resin composition……………………………………………………………………S3
   2.2 Ultraviolet-visible (UV-Vis) Spectroscopy…………………………………………S3
   2.3 Fourier Transform Infrared Spectroscopy (FTIR)…………………………………S4
   2.4 Photo-rheology………………………………………………………………………...S5
3. 3D-printing dual-wavelength resin……………………………………………………S6
   3.1 Dual wavelength negative imaging system (DWNI)………………………………S6
   3.2 Degradation of PμSL printed materials…………………………………………...S7
   3.3 Thermal characterization of PμSL printed materials……………………………....S8
   3.4 Characterization of UV cured materials post-degradation………………………..S9

3.4.1 Effects on surface roughness……………………………………………………S9
3.4.2 Effects on crosslinking density and modulus…...……………………………S11
3.4.3 Effects on resolution …………………………………………………………S12

1. Resin materials

3-Ethyl-3-oxetanemethanol (OXA, >96%), 4-acryloylmorpholine (ACMO, >98%), and diphenyl[4-(phenylthio)phenyl] sulfonium hexafluoro antimonate (DHS, >98%) were purchased from TCI Chemicals. Phenylbis(2,4,6-trimethylbenzoyl) phosphine oxide (BAPO, >97%) and Sudan I (>95%) were purchased from Sigma Aldrich. All chemicals were used as received.

1.1 Methacrylated sebacic acid (MSA) synthesis

Sebacoyl chloride (99%), sodium methacrylate (NaMA, 99%), 4-methoxyphenol (MEHQ, 99%), and acetone (suitable for HPLC, >99.9%) were purchased from Sigma Aldrich and used as received. MSA was synthesized following a previously published procedure with minor modifications.[1] 10 g (41.8 mmol, 1 equiv) of sebacoyl chloride were added to a 250 mL round bottom flask (RBF) with a magnetic stir bar. ~75 mL of reagent grade acetone were added to the RBF under rapid stirring and then 9.05 g (83.6 mmol, 2 equiv) of sodium methacrylate (NaMA) were added to the mixture. 10 mL of reagent grade acetone were used to wash off any remaining NaMA powder on the funnel. The white mixture was allowed to stir at room temperature for 1 hour. Then, the product was transferred into 50 mL Corning tubes and centrifuged at 10,000 RPM for 10 minutes. The supernatant was transferred into a 500 mL RBF and 4.25 mg of MEHQ were added to the product. The filtrate was concentrated into a light yellow viscous product via rotary evaporation to give 13.4 g of the MSA compound (317 ppm MEHQ). The MSA products were stored in foil wrapped glass vials in the -20 °C freezer where they crystallized. Prior to use, MSA products were thawed at room temperature within a few minutes. H-NMR (Fig S1) and 13C-NMR (Fig S2) was used to characterize the product.

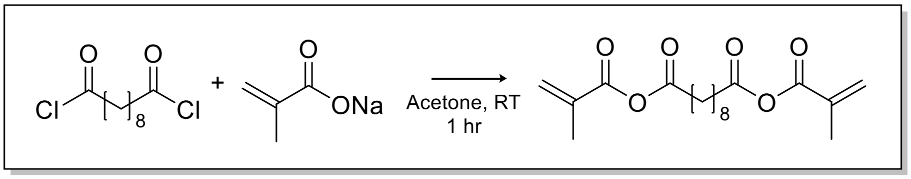

Scheme 1. Synthesis of Methacrylated Sebacic Acid (MSA)

MSA: 1H NMR (500 MHz, $CDCl_3$) δ 6.2 (s, 2H), 5.8 (s, 2H), 2.5 (t, J = 7.4 Hz, 4H), 1.98 (s, 6H), 1.7 (m, J = 7.4 Hz, 4H), 1.42 – 1.3 (m, 9H). δ 2.18 from residual acetone.

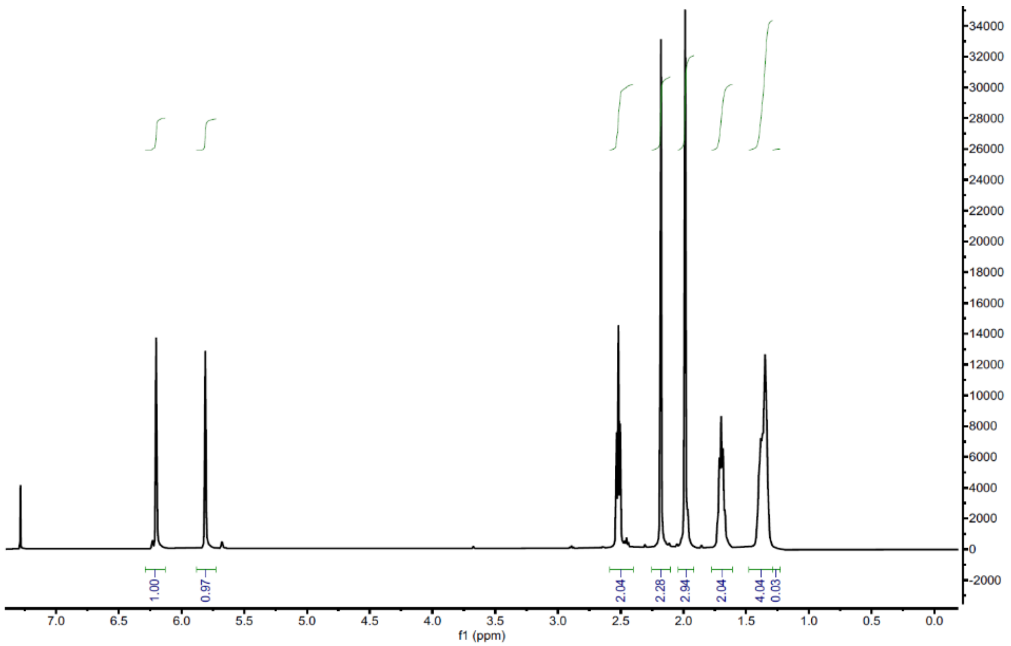

Figure S1. MSA 1-H NMR spectra.

MSA: 13C NMR (125 MHz, $CDCl_3$). δ 169.52, 163.12, 135.75, 128.98, 35.23, 30.93, 24.20, 17.90.

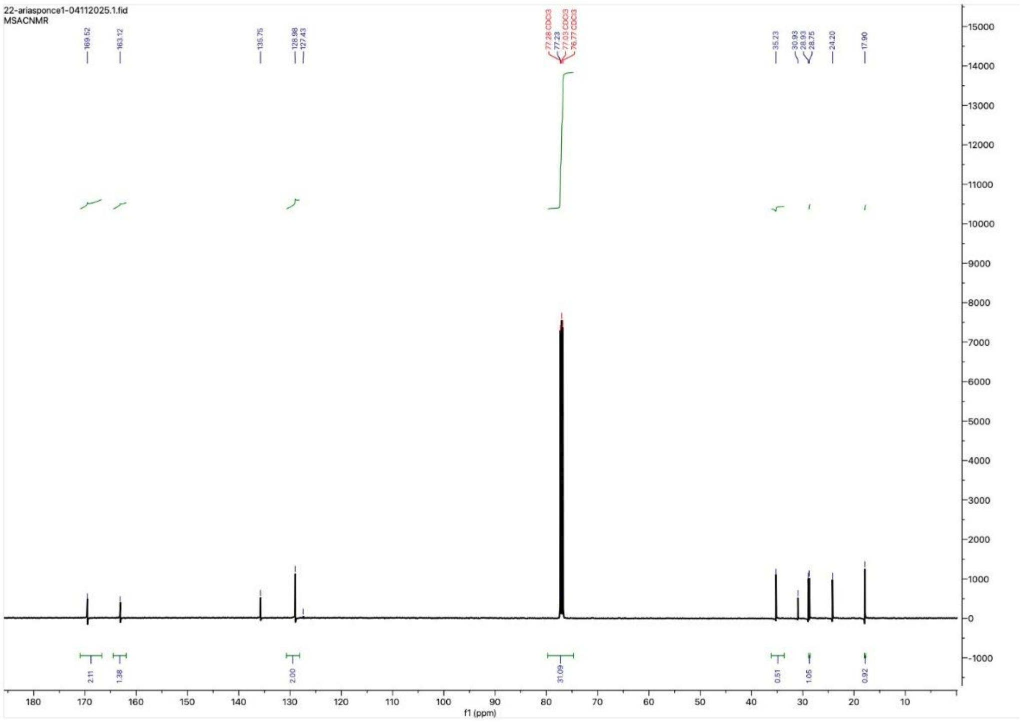

Figure S2. MSA 13C NMR spectra.

2. Dual wavelength resin characterization

2.1 Resin composition

The dual-wavelength, one pot resin was formulated with cationic and radical network components at 80 wt% and 20 wt% loadings, respectively. The cationic network contains epoxy monomers and crosslinkers (OXA, ECC) as well as a photoacid generator (DHS). The radical network contains (meth)acrylate monomers and crosslinkers (ACMO, MSA) as well as a radical photoinitiator (BAPO). MSA was synthesized in a one-step reaction as described in S1.1. Sudan I photoabsorber was integrated at 0.1 wt% loading.

Table S1. Dual wavelength, one pot resin with permanent cationic and degradable radical networks.

	80 wt % (C) - 20 wt % (R)		
**Cationic (C)**	MW (g/mol)	Wt %	Mol %
OXA	116.16	20.80	34.2
ECC	252.3062	56.48	42.7
DHS	607.29	2.72	0.9
Total		80.00	77.78
**Radical (R)**	MW	Wt%	Mol %
MSA	338.17	4.68	2.6
ACMO	141.17	14.05	19.0
BAPO	418.465	1.27	0.6
Total		20.00	22.22

2.2 Ultraviolet-visible (UV-Vis) Spectroscopy

UV-VIS Spectroscopy was performed using a Cary 5000 UV-VIS NIR spectrophotometer (Agilent Technologies) to analyze optical absorbance of resin components, specifically the photoacid generator (DHS) and radical photoinitiator (BAPO). Photoabsorbing components were diluted in acetone to ensure measurements remained within the linear range of the UV-VIS. Samples were placed in quartz cuvettes (Rectangular glass fluorometer cell, Starna Cells Inc) with a path length of 1 cm and absorbance spectra were recorded at a scan rate of 1800 nm/min across the 250-800 nm wavelength range. Backgrounds were subtracted using blank reference samples. The radical photoinitiator (BAPO) absorbed strongly at both 365 nm and 405 nm, as expected (Fig S3). This demonstrates the photoinitiator’s ability to generate radicals at both wavelengths. Conversely, the cationic photoacid generator (DHS) only absorbed under 365 nm (Fig S3). The selectivity of the photoacid to UV light demonstrates its ability to generate acids and radical species at 365 nm wavelength only.

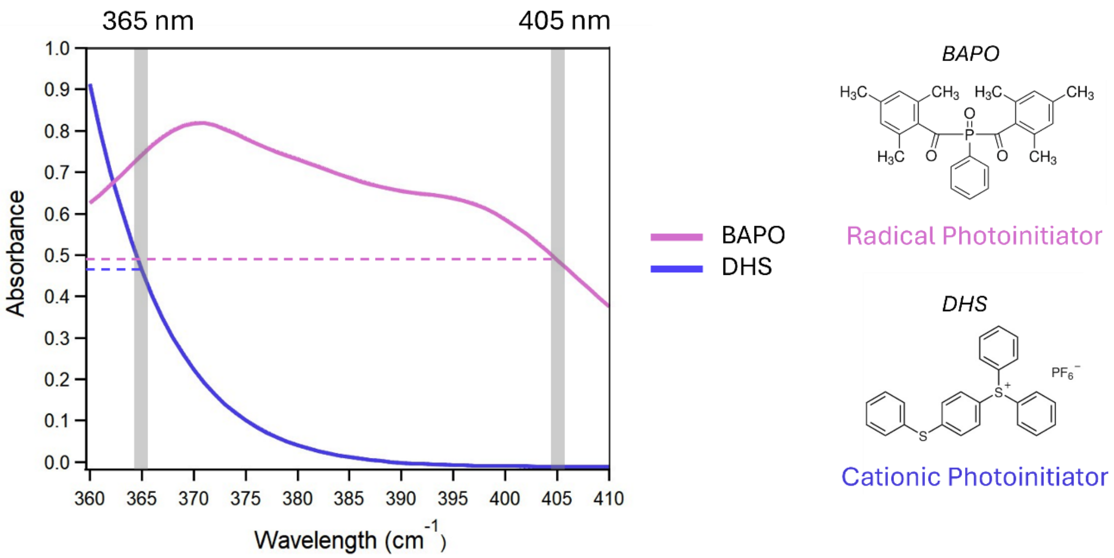

Figure S3. UV-VIS of light-absorbing components show cationic initiator (DHS) absorption at 365 nm and radical initiator (BAPO) absorption at both 365 nm and 405 nm.

2.3 Fourier Transform Infrared Spectroscopy (FTIR) Spectroscopy

The wavelength-selectivity of the cationic and radical polymerizations was also assessed via transmission mode RT-FTIR spectroscopy (Bruker, VERTEX 80) with coupled LEDs at 365 nm (90 mW/cm^2, UHP-F-365, Prizmatix) and 405 nm (30 mW/cm^2, UHP-F-405, Prizmatix). FTIR settings were set for 64s scan time, 64s background scan time, and 4 cm^{-1} resolution. Prior to experiments, light intensity was calibrated for each wavelength at the sample location using a power meter (PM100D, S120VC sensor, Thorlabs). Due to peak convolution, the (meth)acrylate and epoxy network resins were prepared and monitored separately at 20 wt% and 80 wt% concentrations respectively as shown in Table S1. The residual concentration for both resins was completed with propylene carbonate (PPC, >99%, Sigma Aldrich) as it is has low volatility and dissolves solid components. Light was irradiated onto resin samples sandwiched between glass slides after 30 s of initial data collection. The radical polymerization was monitored by observing the disappearance of the C=C double bond band of (meth)acrylate monomers in the NIR region between 6100-6250 cm^{-1}.[2] The cationic polymerization was monitored by tracking the overtones of C-H and CH_2 stretching bands of epoxy monomers in the NIR region 5800-6000 cm^{-1}.[3] The degree of functional group conversion was determined by calculating percent decrease in the integrated areas of (meth)acrylate and epoxy monomer absorption peaks during light irradiation relative to a reference peak before irradiation at either 6100-6250 cm^{-1} for (meth)acrylate monomers or 5800-6000 cm^{-1} for epoxy monomers. Heat post-processing was carried out by placing samples in the oven (ADP300C, Yamato) at 110 °C for 10 and 15 minutes. At those timepoints, epoxy conversion was remeasured via FTIR and increased for 365 nm irradiated samples up to 57% (Fig S4). To improve the final properties of the epoxy network cured at 365 nm, thermal treatment was integrated as a processing step for 3D printed parts going forward. Samples irradiated at 405 nm also showed increased epoxy conversion with heat treatment, however conversion remained low under 30%, thereby preserving degradability (Fig S4).

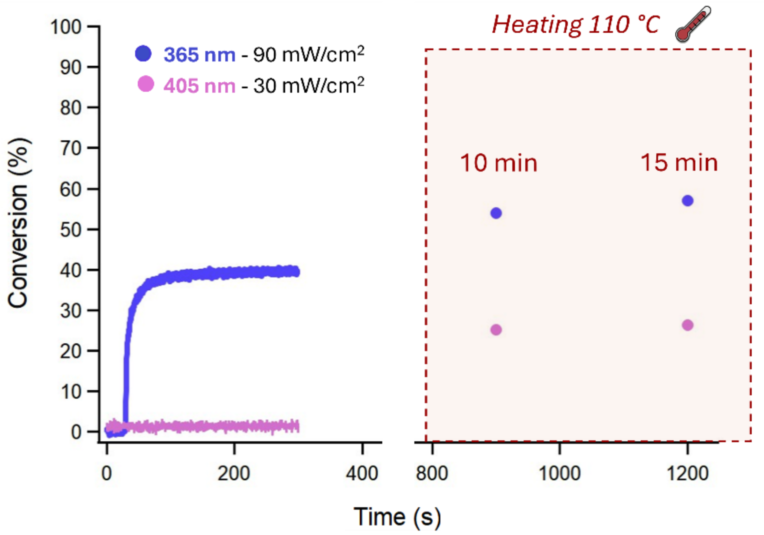

Figure S4. Epoxy monomer conversion increases with heat post-processing at 110 °C.

2.4 Photo-rheology

Photo-rheology measurements were conducted with a TA Instruments Discovery Hybrid Rheometer (DHR-1) equipped with a UV curing accessory and coupled with a light guide to monitor viscoelastic properties during light irradiation under fast oscillation. A disposable aluminum parallel plate geometry (diameter 20 mm) was used with a testing gap of 100 μm with 75 μL of sample volume. Ultra-high powered collimated LED light sources (UFP-F-405 and UHP-F-365, Prizmatix) were used at visible light (405 nm) and UV light (365 nm) wavelengths. Prior to each experiment, light intensity was calibrated using a power meter (PM100D, S120VC sensor, Thorlabs). Light was irradiated onto samples after 30 s of initial data collection. Photorheology results show gelation of the (meth)acrylate network under visible light, while the epoxy network remained in liquid form (Fig S5, left). Conversely, both (meth)acrylate and epoxy networks gelled under UV light, with the epoxy network showing a faster gelation rate and reaching a higher modulus than the (meth)acrylate network (Fig S5, right).

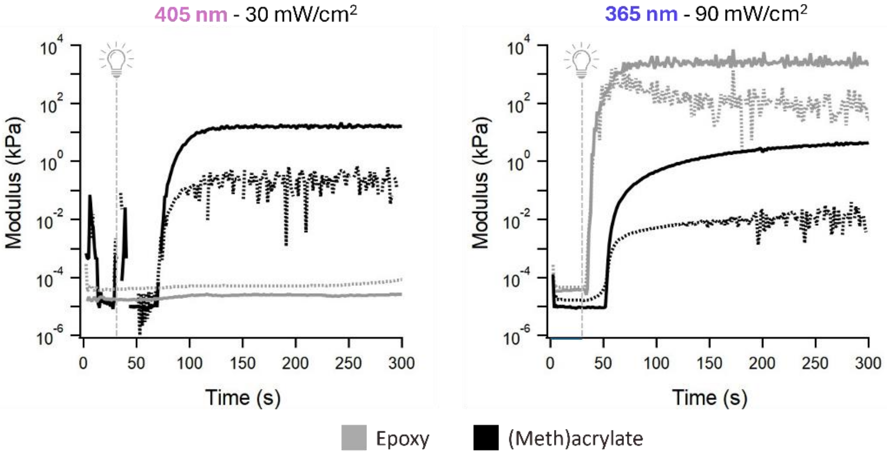

Figure S5. Photorheology shows gelation of (meth)acrylate network under visible light and conversion of both (meth)acrylate and epoxy networks under UV light.

3. 3D-printing dual-wavelength resin

3.1 Dual wavelength negative imaging (DWNI) system

The DWNI system (Fig S6) is a novel type of digital light processing (DLP) 3D printing system, also sometimes called projection micro-stereolithography (PμSL), which utilizes independent 365 nm and 405 nm LED sources (Prizmatix UFP-F-405 and UHP-F-365). Light is delivered via 5mm liquid light guides to two telescopes, which are adjusted for a flat intensity profile. The light from each telescope is then directed to the pixelated digital micromirror device (DMD, DLP9500BFLN, Texas Instruments) which has an array of 1920×1080 square micro-mirrors with a pitch of 10.8µm. The incident beams are mirror image symmetric on the DMD, such that the “off state” beam-path for each wavelength is co-linear with the input beam-path for the other wavelength; this effectively toggles each micromirror to select which wavelength is reflected as the output normal to the DMD plane. This normal-incidence reflected light from the DMD is intended for resin exposure, and is collected by a simple projection microscope consisting of a Thorlabs TL200-UVB tube lens and a Thorlabs long working distance microscope objective. A colinear camera (acA11920-40um, Bassler) is used to view the projected image on the working surface in real time. The system is controlled by the LabVIEW software which controls the DMD, LEDs and the linear XYZ stages (Thorlabs MTS25-Z8). In the software, the National Instruments USBX DAQ (781440-01) is used to control the LEDs on and off times and intensity.

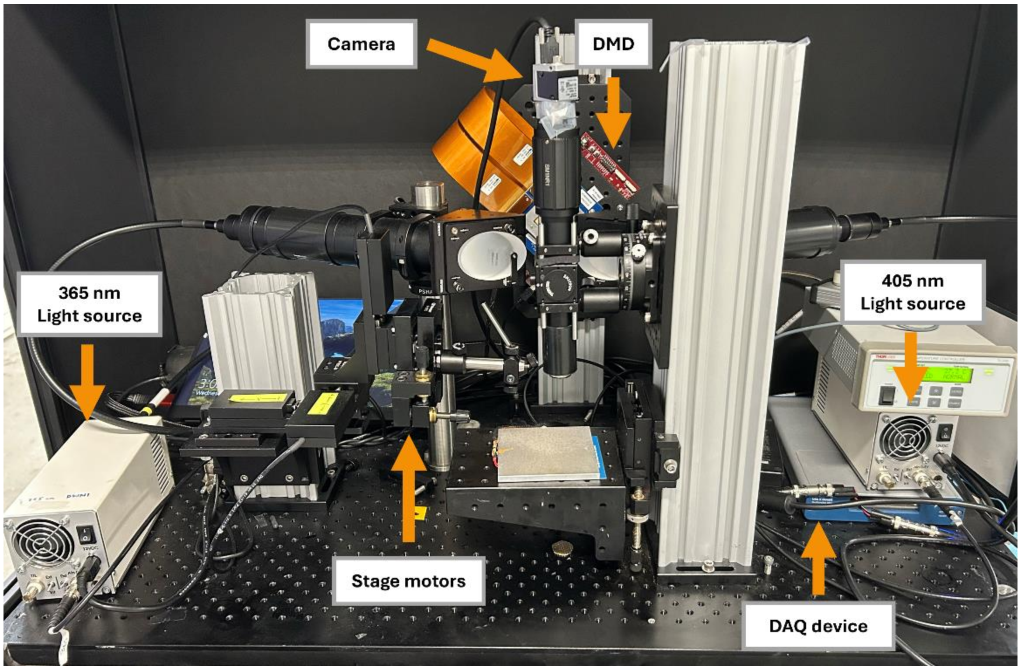

Figure S6. DWNI system as built, highlighting the UV LED sources, DMD with controlled micromirrors, and three-axis stage.

3.2 Degradation of PµSL printed materials

Material degradation was evaluated by exposing discs (3x2mm) fabricated under 365 nm or 405 nm with the dual-wavelength PµSL to a solution of 5M sodium hydroxide (NaOH, >98%, Sigma-Aldrich) or distilled water at room temperature. Discs were dialyzed in isopropyl alcohol and acetone mixtures and thermally treated at 110 °C prior to degradation in solutions. Mass loss was measured at predetermined intervals by drying and weighing the samples. Discs immersed in 5M NaOH solutions degraded within 1.5 hrs (Fig S7) and discs immersed in water degraded within 18 hrs (Fig S8). Only the (meth)acrylated anhydride crosslinker is susceptible to base degradation, thus leaving the majority epoxy network (80 wt%) physically and chemically unaffected following base treatment. This was demonstrated in Fig S7, which shows that mass loss for 365 nm cured materials was equivalent to ~20 wt% after 3 hrs of degradation in 5M NaOH solution. We anticipate that the mass loss of the methacrylated network accounts for 20 wt% porosity in the final epoxy parts.

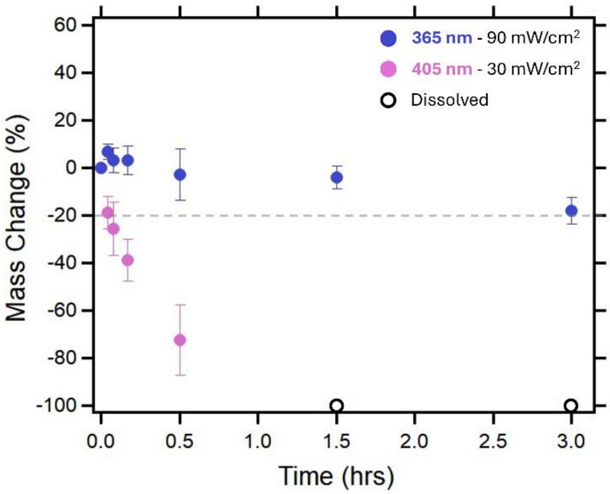

Figure S7. Mass loss data for 405 nm and 365 nm irradiated materials in 5M NaOH solution.

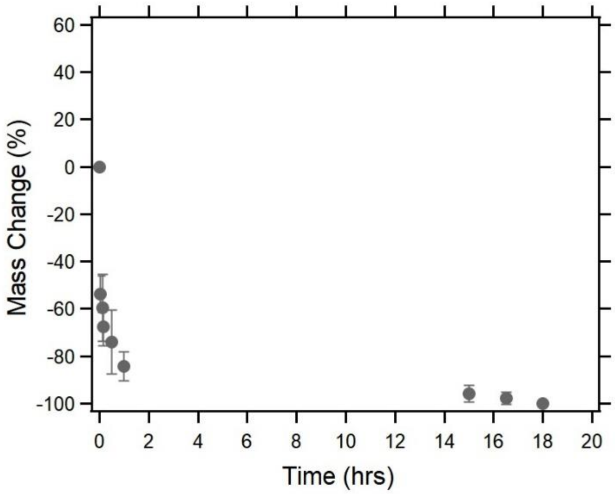

Figure S8. Mass loss data for 405 nm irradiated materials in water.

3.3 Thermal characterization of PμSL printed materials

Differential scanning calorimetry (DSC) was performed using a Discovery DSC 2500 (TA Instruments) with a heat-cool-heat cycle to evaluate the thermal properties of the cured thermoset network. Visible light cured discs (3x2 mm) were printed in the dual-wavelength PμSL, dialyzed in isopropanol and acetone mixtures, and dried in the oven at 80 °C for 1 hr. Dried samples (~8 mg) were loaded onto Tzero Aluminum pans. Following the heat-cool-heat procedure, samples were first heated from room temperature to 200 °C at a rate of 10 °C/min, followed by cooling to -100 °C at 5 °C/min, and then reheated under the initial conditions. The glass transition temperature (Fig S9) was determined from the second heating cycle to eliminate thermal history. Data processing was performed with Trios software (TA Instruments). The DSC data shows a broad glass transition peak for both networks, with 365 nm (UV-cured) networks showing a higher glass transition (T_g ~ 101°C) than 405 nm (visible-light) cured networks (T_g ~ 67°C).

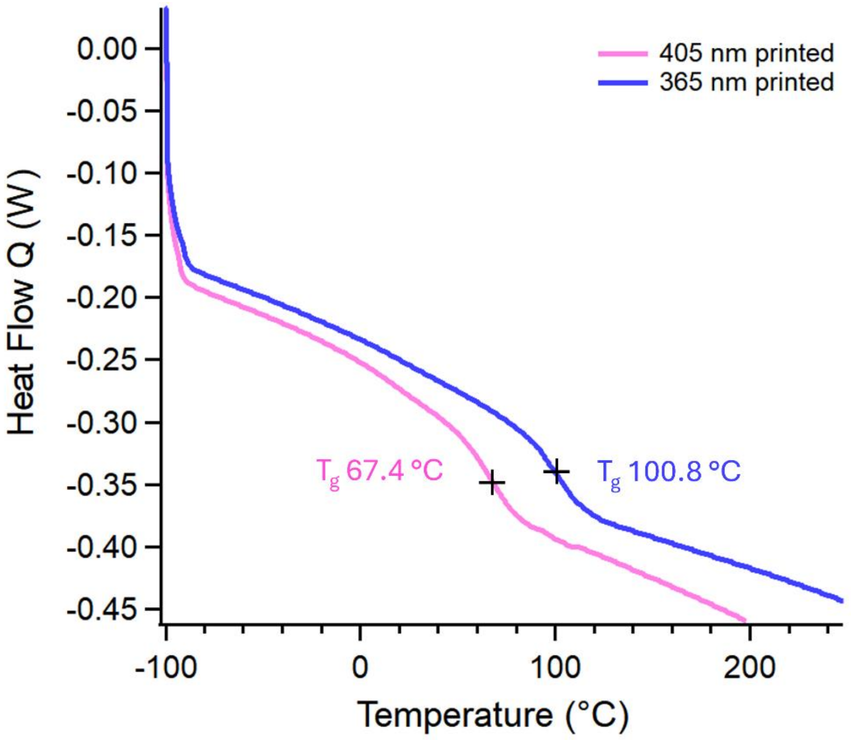

Figure S9. Differential scanning calorimetry (DSC) data for 405 nm and 365 nm cured discs.

3.4 Characterization of UV cured materials post-degradation

3.4.1 Effects on surface roughness

We obtained microscope images of the 365 nm cured parts before and after the degradation process (Fig S10) with a Keyence VHX digital microscope. From the top view, the degraded parts appeared smoother and more sharply defined possibly due to the removal of unspecific cured regions formed by scattered light. In contrast, the side view at high magnification shows layer-to-layer discontinuities which may arise from epoxy conversion gradients along the z-axis. Regions that are less cured with 365 nm light are more susceptible to dissolution, which creates a stepped appearance between cured layers and voids in between.

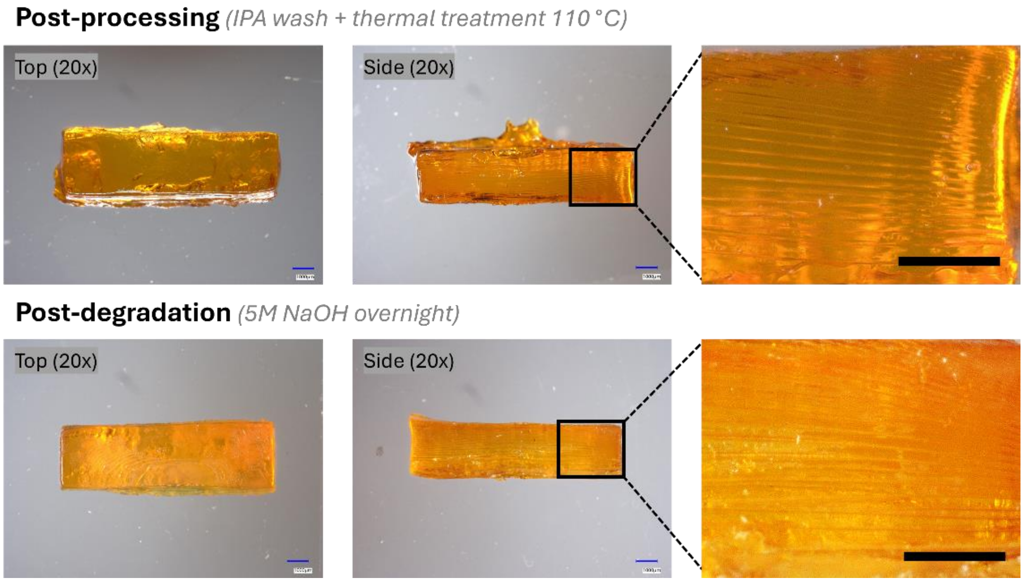

Figure S10. Microscope images of 365 nm-cured parts after post-processing (top row) and post-degradation (bottom row). Side views (20, 80x) show layer-to-layer discontinuities. Scale bars are 1 mm.

At higher magnification on the side view, we extracted line surface roughness profiles both across multiple layers (Figure S11B, S11D) and across a single layer (Figure S11A, S11C) after post-processing and base degradation steps with the Keyence VHX depth from defocus (DFD) 3D profiling. The surface roughness profile along a single layer showed a relatively homogenous layer pre- (S11A) and post-degradation (S11C) with a variability of ~12-14 µm. In contrast, the surface roughness profile across multiple layers showed higher levels of variability after base degradation of ~40 µm (Figure S11D). Future work to address the discontinuity artifacts could include optimizing print parameters by using smaller layer thicknesses to improve epoxy conversion and uniformity between layers.

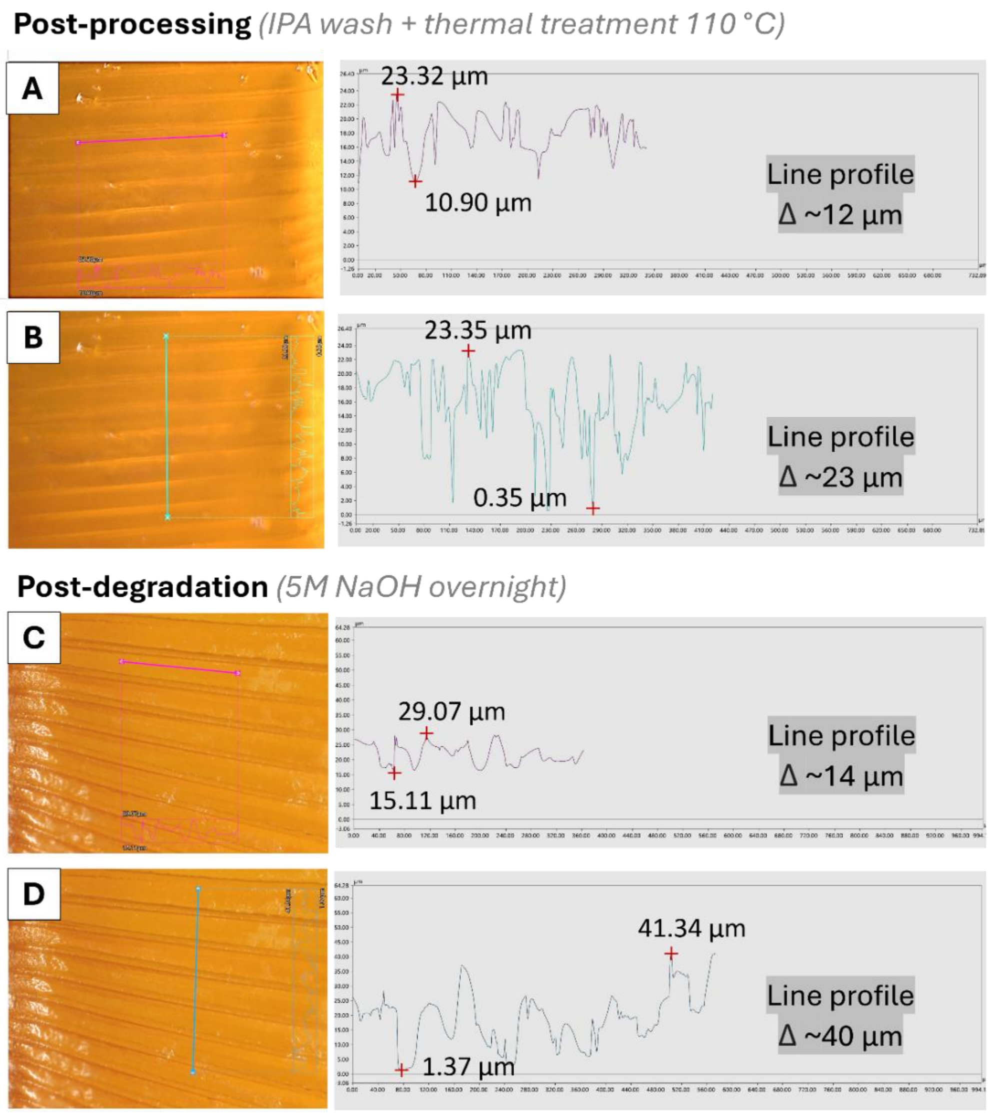

Figure S11. Surface roughness profiles measured by Keyence VHX microscope depth from defocus (DFD) profiling both across multiple layers and along a single layer after post-processing steps (A, B) and post-degradation with 5M NaOH (C, D). The surface roughness profile across multiple layers (B, D) showed higher levels of variability post-degradation, while the surface roughness profile along a single layer (A, C) showed similar variability before and after degradation.

3.4.2 Effects on crosslinking density and modulus

Although some microporosity forms in the UV-cured parts post-degradation, the overall mechanical strength and stiffness of the final prints remain governed by the robust epoxy network. Dynamic mechanical analysis (DMA) of the parts before degradation shows a rubbery modulus of 2.69×10^7 Pa at 200 °C, corresponding to a crosslink density of approximately 2278 mol/m^3 (Fig

S11). After base-mediated degradation of the (meth)acrylate network, the modulus slightly decreases to 1.74 × 10^7 Pa, reflecting a crosslink density of 1474 mol/m³ (Fig S12). A modest decrease in glass transition temperature is also observed, from 152.1 °C to 139.9 °C, further indicating partial loss of the degradable network. Despite a ~35% reduction in the crosslink density, the bulk thermomechanical performance is largely preserved, indicating that the permanent epoxy network continues to dominate the mechanical properties of the printed parts even after removal of the degradable network. Due to the limited projection area of the DWNI printer, samples with suitable length for DMA testing were prepared by casting the resin into silicone molds. The resin was cured under 365 nm LED light with exposure energy matched to the printing process to ensure comparable network structure.

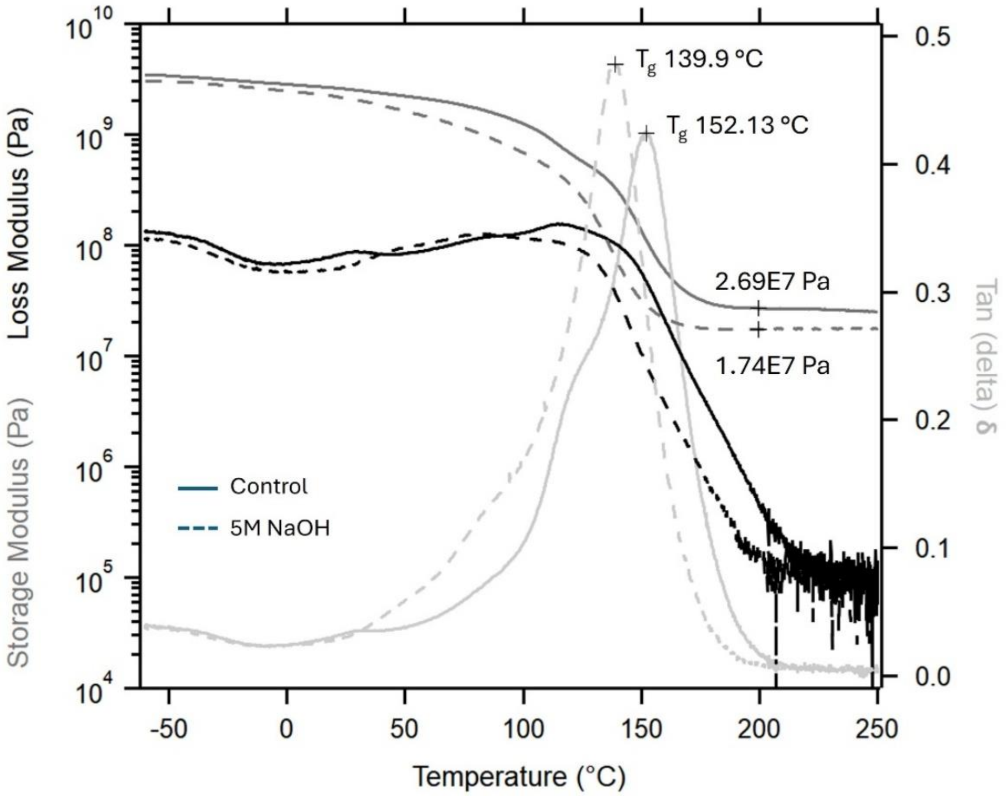

Figure S12. Dynamic mechanical analysis (DMA) curves of printed parts before (solid line) and after (dotted line) degradation.

3.4.3 Effects on resolution

The selective degradation of the (meth)acrylate network by the base treatment enhances resolution and dimensional accuracy by eliminating unintended crosslinking in areas affected by scattered light exposure (Fig S13, top and middle). In addition, exposure to both visible and UV light appears to improve feature resolution (smallest feature: 210 µm; Fig S13, bottom) and print fidelity

compared to UV-only exposure (smallest feature: 260 μm; Fig S13, middle). This improvement may arise from the formation of the degradable (meth)acrylate network that temporarily constrains the epoxy network during curing, allowing for more precise shape retention and higher dimensional accuracy.

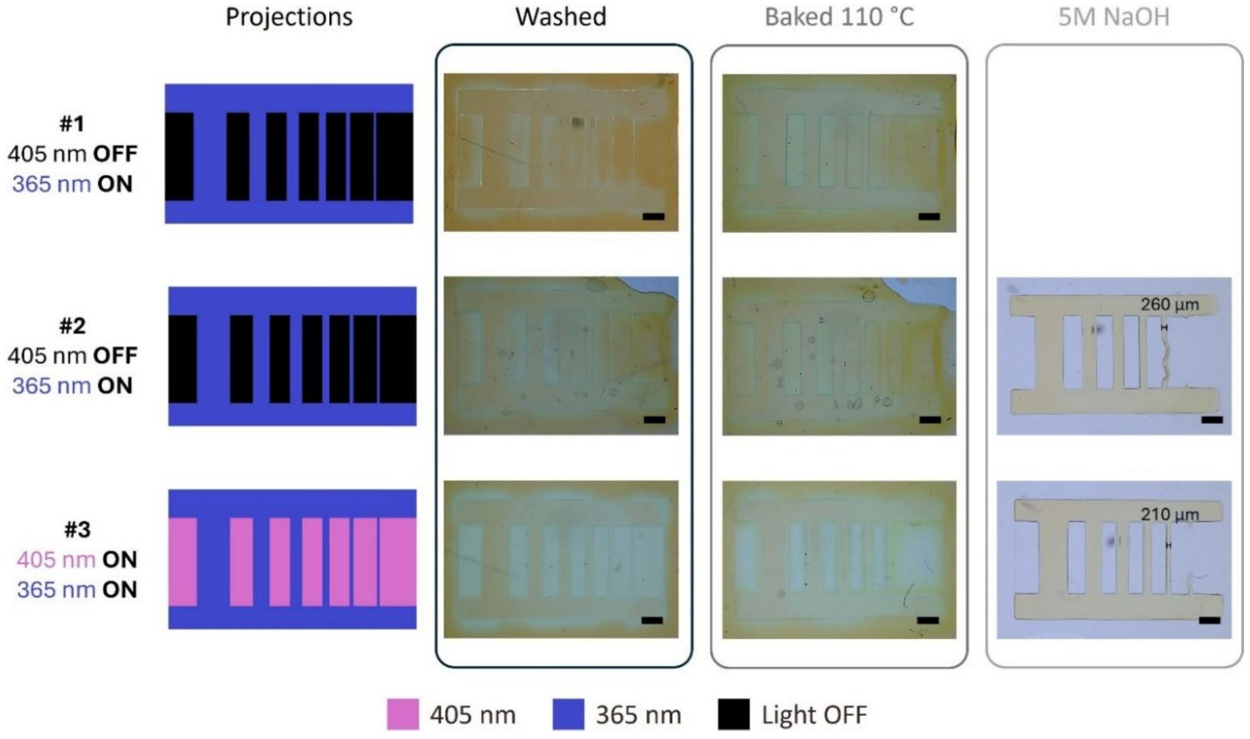

Figure S13. Resolution tests showing UV cured patterns under varying conditions, with (1) 405 nm OFF and 365 nm ON without post-degradation, (2) 405 nm OFF and 365 nm ON with post-degradation, and (3) 405 nm ON and 365 nm ON with post-degradation.

References

(1) Bagnall, N. R.; Jones, M. H.; Jernigan, G. C.; Routt, C.; Dar, L. C.; Worrell, B. T. Catalytic, Sulfur-Free Chain Transfer Agents That Alter the Mechanical Properties of Cross-Linked Photopolymers. *J. Am. Chem. Soc.* **2023**, *145* (26), 14202–14207. https://doi.org/10.1021/jacs.3c03811.

(2) Launay, V.; Wolf, R.; Dumur, F.; Lalevée, J. Photothermal Activation in the near Infrared Range for 4-Dimensional Printing Using Relevant Organic Dyes. *Addit. Manuf.* **2022**, *58*, 103031. https://doi.org/10.1016/j.addma.2022.103031.

(3) González, M. G.; Cabanelas, J. C.; Baselga, J. Infrared Spectroscopy - Materials Science, Engineering and Technology. **2012**. https://doi.org/10.5772/36323.
