# Supplementary material for: Dual-Wavelength Simultaneous Patterning of Degradable Thermoset Supports for One-Pot Embedded 3D Printing
Source: ACS Cent Sci. 2025 Jun 4;11(6):967–74. doi: 10.1021/acscentsci.5c00337 (PMC12203427; doi:10.1021/acscentsci.5c00337)
Supplement: Supplementary file 3 [file oc5c00337_si_003.pdf]

oc-2025-003379.R1

Name: Peer Review Information for "Dual-wavelength simultaneous patterning of degradable thermoset supports for one-pot embedded 3D printing"

First Round of Reviewer Comments

Reviewer: 1

Comments to the Author

Review

The presented manuscript is a well-prepared and interesting new strategy for 3D printing, which is of interest to the ACS Central Science readership. However, the authors must state how they differentiate themselves from a preprint published in Dec 2024 prior to publication (see first question). Additionally, the role of varying crosslinking density due to grayscale printing (different exposure at the two wavelengths) should be illuminated more critically in a revised version. Some additional, mostly minor remarks are also outlined below.

Major comments:

1. The authors should further mention the following preprint and differentiate their work from it: https://chemrxiv.org/engage/chemrxiv/article-details/67570ef57be152b1d06c7cbb

2. In the introduction, the authors should include two modern photopolymerization-based 3D printing techniques and their significance in the context of the presented work: Volumetric 3D printing and two-/multi-photon polymerization based 3D printing techniques also do not require support structures to print unconnected objects within a cage, overhangs, etc..

On the basis of this, the following two comments at the end of the Results and Conclusion sections should be revised:

a) “These objects would be challenging to fabricate with traditional additive manufacturing methods as they are not structurally supported which demonstrates the advantage of the embedded DWNI DLP printing approach.”

b) “Our dual wavelength DLP printing strategy enables the facile fabrication of free-floating and unsupported geometries that would be impossible to achieve with traditional manufacturing methods.”

3. Why did the authors test a different range of exposure range for 365 nm vs 405 nm irradiation? Is this an instrumental limitation? Since non-degradable scaffolds could only be produced from high exposures on at 365 nm, the authors should prove that degradation of the scaffolds is still possible for the same exposure values at 405 nm. However, this range is not probed for the 405 nm irradiated samples. Therefore, the authors cannot rule out that greyscale printing effects (= effects due to printing at different intensities) are, at least partially, responsible for the degradable/non-degradable effects.

4. Thank you for a very nice SI – I very much appreciate it!

Minor comments:

5. Please clarify under which conditions the comment that print time is reduced to 50% is justified: only if support structures as elaborate as used in this work are actually required. In this work, entire gels are printed around the object as a result of the negative imaging strategy, which would not be the case if traditional support structures were designed. Additionally, one could argue that exposure times required to pattern non-degradable features are much longer than exposure times required to simply produce a green body for post-curing if traditional supports were used.

6. Please add a DSC for the UV-cured system in Figure S8 for comparison.

7. Could the authors please comment on the potential for variability in the resin composition for future application?

8. “Base selectively degrades regions with lower epoxy conversion, predominantly those away from the light source, where lower UV exposure results in insufficient cationic polymerization.” – Please clarify: what is meant by “predominantly those away from the light source”?

9. Figure 4B: please add a legend for the symbols used in the graphs.

10. The nice supplementary video should be mentioned in the main paper.

Reviewer: 2

Comments to the Author

Ponce and co-workers present here a one-pot embedded DLP 3D printing method combining degradable thermoset supports and dual-wavelength photocuring strategy. Based on the fact that degradable methacrylate crosslinkers work under visible light (405 nm) via radical polymerization, while the non-degradable epoxy monomers polymerize under UV (365 nm) light only, the 3D printing process realizes multimaterial fabrication, and the degradable part can be removed after printing under high temperature in alkaline solutions. The proposed approach is a smart combination of chemical design and advanced 3D printing technqiues, which achieved the in situ fabrication of complex structures with sacrificing support structures. Despite the interesting work, some critical points should be considered:

1. Dual-wavelength 3D printing technique is not a new concept, and it has been extensively explored in last 5-10 years for multimaterial vat photopolymerization, by several significant research groups in the field. During this process, both photoinhibitor and photoswitch based approaches have been developed, which realized volumetric printing without the need of support structures. On the other hand, tomographic reconstruction has also been invented to achieve the same goal. Compared to these methods, the current work has not shown convincing advantages. In addition to that, the authors did not even discuss these works and do a comparison.

2. The next question is how the degradation step would influence the final material performance of the 3D printed structures. The reviewer can see two significant aspects: the internal structures of the 3D printing networks and the mechanical properties. After the degradation and washing away, how the porosity and crosslinking density can change? Consequently, how this would affect the mechanical strength and modulus of the 3D printing products? These are very important parameters for the eventual application of the 3D printing materials. However, they are apparently neglected.

Moreover, how is the resolution and surface smoothness can be influenced? A more thorough study may be needed, regarding that numerous advanced volumetric and dual-wavelength 3D printing techniques have been developed with great merits already.

For molecular synthesis, 13C NMR and HRMS were not provided.

Author's Response to Peer Review Comments:

Dear Editor,

Thank you for forwarding the reviewer comments. We have addressed all the feedback thoroughly, with the corresponding changes clearly highlighted in the revised manuscript.

Given the unique material design and broad applicability of our system, we anticipate strong interest from the journal's readership. We believe that our dual-wavelength, one-pot resin system represents a significant advancement in unsupported 3D printing and is well-suited for publication in this high-impact, multidisciplinary journal.

We appreciate your consideration and look forward to your response.

Sijia

Authors' Responses to Reviewers:

Reviewer: 1

Review
The presented manuscript is a well-prepared and interesting new strategy for 3D printing, which is of interest to the ACS Central Science readership. However, the authors must state how they differentiate themselves from a preprint published in Dec 2024 prior to publication (see first question). Additionally, the role of varying crosslinking density due to grayscale printing (different exposure at the two wavelengths) should be illuminated more critically in a revised version. Some additional, mostly minor remarks are also outlined below.

Major comments:

1. The authors should further mention the following preprint and differentiate their work from it: https://chemrxiv.org/engage/chemrxiv/article-details/67570ef57be152b1d06c7cbb

Author reply:

We thank the reviewer for pointing out this relevant preprint from Diaco and coworkers at MIT. We have referenced it in the main manuscript at the end of manuscript page 4.

The key differences between that work and ours, are as follows: the MIT team developed a dual-wavelength resin to fabricate multimaterial objects with dissolvable regions using a thermoplastic-based network for the removable supports. Complementary to that work, we have developed a dual-wavelength resin using a thermoset network to fabricate the degradable supports. While their degradable material is composed of an isobornyl acrylate (IBOA) based thermoplastic network that dissolves in organic solvent, our degradable material consists of a methacrylated sebacic acid (MSA) crosslinker and acryloyl morpholine (ACMO) comonomer to form a thermoset network that degrades under basic aqueous conditions. By solidifying at lower concentrations than thermoplastics, our thermoset degradable network allows a greater fraction of the material to comprise the permanent epoxy network, in our case doubling to 80 wt% compared to the 40 wt% achieved by Diaco and coworkers in a one-pot system, maximizing the thermomechanical properties of the final parts. In addition, we use a custom-built dual-wavelength negative imaging (DWNI) 3D printer that employs a single digital micromirror device to project both wavelengths of light. This design is different from commercial systems, which typically align two digital micromirror devices or can only project light sources sequentially.

2. In the introduction, the authors should include two modern photopolymerization-based 3D printing techniques and their significance in the context of the presented work: Volumetric 3D printing and two-/multi-photon polymerization based 3D printing techniques also do not require support structures to print unconnected objects within a cage, overhangs, etc..
On the basis of this, the following two comments at the end of the Results and Conclusion sections should be revised:

We thank the reviewer for pointing out this gap in our introduction. We have introduced volumetric 3D printing and multi-photon approaches with brief descriptions about these technologies as well as limitations that could be addressed via dual-wavelength approaches as shown below:

"Some VP approaches have been developed which allow support-free printing. For example, tomographic volumetric additive manufacturing (VAM) enables layer-less fabrication of complex objects by exposing resin to light projections from multiple angles.[21,22] While it relies on a unique hardware configuration, custom optics, and advanced software algorithms to deliver controlled light dosages, it can build entire objects in a single, continuous exposure step, significantly enhancing throughput. A more well-developed support-free VP technology is two-photon or multi-photon printing (we refer to these collectively as 2PP), which enables support-free structure fabrication by confining polymerization to a sub-micron volume in which two or more photons are absorbed simultaneously.[23,24] Despite their geometric versatility and high resolution, 2PP processes are typically low throughput and have limited build volumes, making them more suitable for high-precision and small-scale applications.

In this context, eliminating the need to add supports in layered VP technologies can be impactful primarily because SLA and DLP printing has gained commercial adoption across multiple industries. These technologies are more broadly accessible than VAM or 2PP, requiring fewer specialized optical components than VAM and allowing for larger build volumes than 2PP. Introducing dual-wavelength multimaterial resins to enable SLA or DLP printing of degradable supports reduces the barrier to fabricating complex, support-free objects."

Citations:

21. Kelly BE, Bhattacharya I, Heidari H, et al. Volumetric additive manufacturing via tomographic reconstruction. Science 2019;363(6431):1075–1079; doi: 10.1126/science.aau7114.

22. Shusteff M, Browar AEM, Kelly BE, et al. One-step volumetric additive manufacturing of complex polymer structures. Sci Adv 2017;3(12):eaao5496; doi: 10.1126/sciadv.aao5496.

23. O'Halloran S, Pandit A, Heise A, et al. Two-Photon Polymerization: Fundamentals, Materials, and Chemical Modification Strategies. Adv Sci 2023;10(7):2204072; doi: 10.1002/advs.202204072.

24. Geng Q, Wang D, Chen P, et al. Ultrafast multi-focus 3-D nano-fabrication based on two-photon polymerization. Nat Commun 2019;10(1):2179; doi: 10.1038/s41467-019-10249-2.

a) "These objects would be challenging to fabricate with traditional additive manufacturing methods as they are not structurally supported which demonstrates the advantage of the embedded DWNI DLP printing approach."

We have revised this sentence to:

“These objects would be challenging to fabricate with layer-based additive manufacturing VP printing methods as they are not structurally supported by previous layers which demonstrates the advantage of the embedded DWNI DLP printing approach.”

b) “Our dual wavelength DLP printing strategy enables the facile fabrication of free-floating and unsupported geometries that would be impossible to achieve with traditional manufacturing methods.”

“Our dual wavelength DLP printing strategy enables the facile fabrication of disconnected or unsupported geometries that would be difficult impossible to achieve at this scale with traditional layer-based manufacturing methods.”

3. Why did the authors test a different range of exposure range for 365 nm vs 405 nm irradiation? Is this an instrumental limitation? Since non-degradable scaffolds could only be produced from high exposures on at 365 nm, the authors should prove that degradation of the scaffolds is still possible for the same exposure values at 405 nm. However, this range is not probed for the 405 nm irradiated samples. Therefore, the authors cannot rule out that greyscale printing effects (= effects due to printing at different intensities) are, at least partially, responsible for the degradable/non-degradable effects.

We tested different light intensities for 365 nm and 405 nm to achieve the strongest contrast in terms of degradability and non-degradability at a constant layer time. Based on UV/VIS screening of our photo initiating components, the photoacid generator that drives the cationic non-degradable reaction does not absorb at 405 nm light, it only absorbs in the UV range at 365 nm. Even at high powers of 405 nm light (90 mW/cm^2), to match 365 nm power (90 mW/cm^2), we did not expect the cationic reaction to be triggered. Therefore, grayscale-induced effects from different light intensities would not be accountable for the degradable/non-degradable contrast.

To further demonstrate this point, we included a figure below which shows a cured square feature with 405 nm light at high power (90 mW/cm^2). After post-processing steps and base degradation, the cured feature remained degradable and completely disintegrated within 10 min. This shows that removing greyscale effects doesn’t impact the degradable/non-degradable contrast between different light sources.

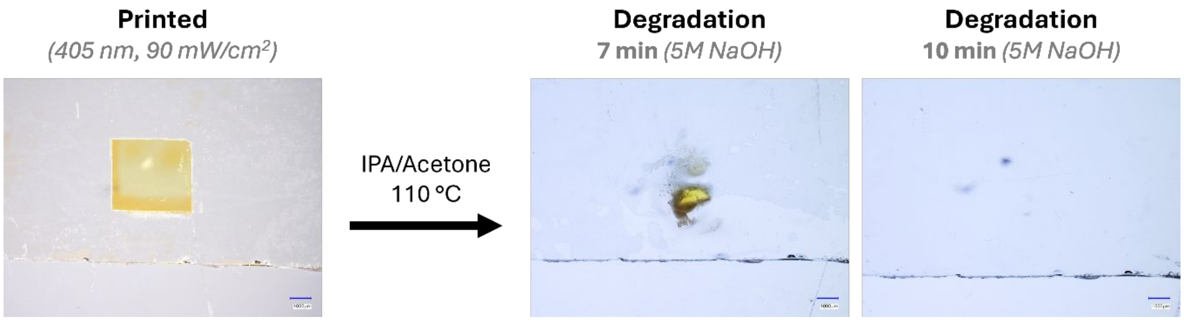

Figure 1. A square feature cured at 405 nm (90 mW/cm², 70s) undergoes post-processing and base degradation, showing partial degradation within 7 min and complete disintegration within 10 min.

4. Thank you for a very nice SI – I very much appreciate it!

Minor comments:

5. Please clarify under which conditions the comment that print time is reduced to 50% is justified: only if support structures as elaborate as used in this work are actually required. In this work, entire gels are printed around the object as a result of the negative imaging strategy, which would not be the case if traditional support structures were designed. Additionally, one could argue that exposure times required to pattern non-degradable features are much longer than exposure times required to simply produce a green body for post-curing if traditional supports were used.

We have clarified in the manuscript that our custom made dual-wavelength negative imaging (DWNI) 3D printer reduces layer time up to 50% by simultaneously projecting both light sources with a single digital micromirror device, specifically in cases where the same layer time is allocated to each wavelength. In conventional dual-wavelength approaches, printers perform two sequential exposures per layer, effectively doubling the print time.

For DLP systems, print times are dictated by the allocated exposure time per layer, regardless of whether we pattern entire embedded volumes or small targeted supports independently of the complexity of the layer. Therefore, embedding the entire gel around the final object in our negative imaging strategy does not increase the print time as it is achieved via a single projection per layer.

We agree with the reviewer that exposure times required to pattern non-degradable features could be longer than those needed to produce a green body and we have acknowledged this in our discussion. However, prolonged thermal post-curing of a green body to fully convert the network could allow monomers to migrate between degradable and non-degradable regions, reducing resolution and compromising the degradability of supports.

Alternatively, using traditional supports which are manually removed could be challenging with stiff and brittle epoxy networks like those in our study. Mechanical stress from the manual removal

of supports could generate cracks or stress concentrators. In contrast, our strategy preserves the integrity of the final structures by removing supports under aqueous conditions.

6. Please add a DSC for the UV-cured system in Figure S8 for comparison.

We have attached an updated DSC curve for both the visible-light and UV-cured networks in Figure S8. The DSC data shows a broad glass transition peak for both networks, with 365 nm (UV-cured) network showing a higher glass transition (T_g ~ 101°C) than 405 nm (visible-light) cured networks (T_g ~ 67°C).

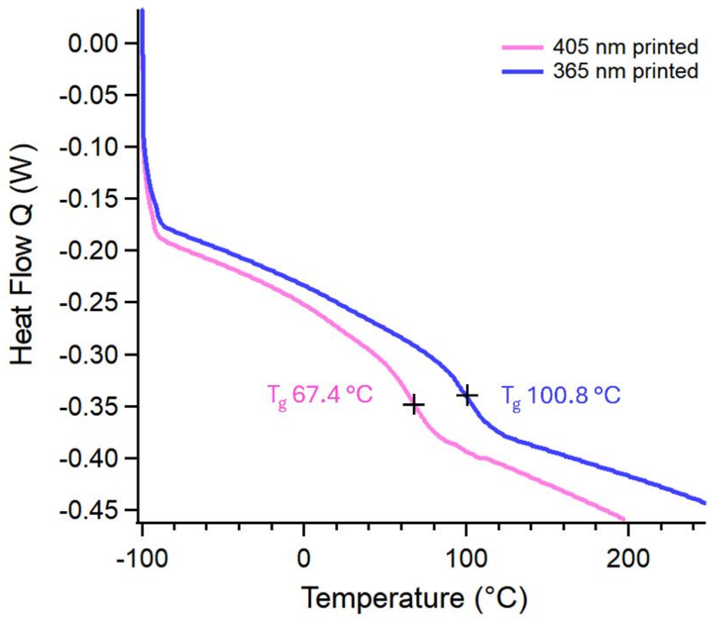

Figure S8. Differential scanning calorimetry (DSC) data for 405 nm and 365 nm cured discs.

7. Could the authors please comment on the potential for variability in the resin composition for future application?

We have added the following text to the discussion at the end of Section 2.2:

"While thermal treatment improved thermomechanical properties, it may also compromise resolution due to monomer diffusion between degradable and non-degradable regions which may result in epoxy conversion in the visible light cured, degradable regions. Therefore, introducing photosensitizers with high absorptivity in the UV region (365 nm), such as 3,6-dimethoxy-9H-thioxanthen-9-one (MeOTX), into the resin composition could enhance the efficiency of photoacid generators and potentially reduce the need for thermal post-curing. In addition, varying the OXA/ECC monomer concentration could be help enhance epoxy conversion rates, allowing for shorter layer times, and increasing print efficiency."

8. "Base selectively degrades regions with lower epoxy conversion, predominantly those away

from the light source, where lower UV exposure results in insufficient cationic polymerization." – Please clarify: what is meant by "predominantly those away from the light source"?

We have changed the sentence to the one below:

"Regions furthest from the light source and deeper into the resin are subject to higher resin absorption and scattering events which attenuate light. This results in lower UV exposures and insufficient cationic polymerization. These regions therefore have overall lower epoxy conversions and are more susceptible to degradation with base."

This explains why the cured features in Fig 4A (top) and Fig 4B (left) shrink in layer thickness after base treatment. The regions closest to the glass substrate (nearest to the light source) survive while those farther away (deeper into the resin) degrade.

9. Figure 4B: please add a legend for the symbols used in the graphs.

We have added legends to the Figure.

10. The nice supplementary video should be mentioned in the main paper.

Thank you for letting us know. We have mentioned the video in the manuscript Section 2.5.

Reviewer: 2

Comments:

Ponce and co-workers present here a one-pot embedded DLP 3D printing method combining degradable thermoset supports and dual-wavelength photocuring strategy. Based on the fact that degradable methacrylate crosslinkers work under visible light (405 nm) via radical polymerization, while the non-degradable epoxy monomers polymerize under UV (365 nm) light only, the 3D printing process realizes multimaterial fabrication, and the degradable part can be removed after printing under high temperature in alkaline solutions. The proposed approach is a smart combination of chemical design and advanced 3D printing techniques, which achieved the in situ fabrication of complex structures with sacrificing support structures. Despite the interesting work, some critical points should be considered:

1. Dual-wavelength 3D printing technique is not a new concept, and it has been extensively explored in last 5-10 years for multimaterial vat photopolymerization, by several significant research groups in the field. During this process, both photoinhibitor and photoswitch based approaches have been developed, which realized volumetric printing without the need of support structures. On the other hand, tomographic reconstruction has also been invented to achieve the

same goal. Compared to these methods, the current work has not shown convincing advantages. In addition to that, the authors did not even discuss these works and do a comparison.

We thank the reviewer 2 for pointing out these gaps in our introduction which were also noted by reviewer 1. As suggested by both reviewers, we have referenced in our introduction both volumetric 3D printing and multi-photon approaches, which are both vat photopolymerization techniques which enable the fabrication of supportless geometries. We have included brief descriptions about each of these technologies as well as limitations that could be addressed via dual-wavelength approaches as shown below:

"Some VP approaches have been developed which allow support-free printing. For example, tomographic volumetric additive manufacturing (VAM) enables layer-less fabrication of complex objects by exposing resin to light projections from multiple angles.[21,22] While it relies on a unique hardware configuration, custom optics, and advanced software algorithms to deliver controlled light dosages, it can build entire objects in a single, continuous exposure step, significantly enhancing throughput. A more well-developed support-free VP technology is two-photon or multi-photon printing (we refer to these collectively as 2PP), which enables support-free structure fabrication by confining polymerization to a sub-micron volume in which two or more photons are absorbed simultaneously.[23,24] Despite their geometric versatility and high resolution, 2PP processes are typically low throughput and have limited build volumes, making them more suitable for high-precision and small-scale applications.

In this context, eliminating the need to add supports in layered VP technologies can be impactful primarily because SLA and DLP printing has gained commercial adoption across multiple industries. These technologies are more broadly accessible than VAM or 2PP, requiring fewer specialized optical components than VAM and allowing for larger build volumes than 2PP. Introducing dual-wavelength multimaterial resins to enable SLA or DLP printing of degradable supports reduces the barrier to fabricating complex, support-free objects."

Citations:

21. Kelly BE, Bhattacharya I, Heidari H, et al. Volumetric additive manufacturing via tomographic reconstruction. Science 2019;363(6431):1075–1079; doi: 10.1126/science.aau7114.

22. Shusteff M, Browar AEM, Kelly BE, et al. One-step volumetric additive manufacturing of complex polymer structures. Sci Adv 2017;3(12):eaao5496; doi: 10.1126/sciadv.aao5496.

23. O'Halloran S, Pandit A, Heise A, et al. Two-Photon Polymerization: Fundamentals, Materials, and Chemical Modification Strategies. Adv Sci 2023;10(7):2204072; doi: 10.1002/advs.202204072.

24. Geng Q, Wang D, Chen P, et al. Ultrafast multi-focus 3-D nano-fabrication based on two-photon polymerization. Nat Commun 2019;10(1):2179; doi: 10.1038/s41467-019-10249-2.

2. The next question is how the degradation step would influence the final material performance of the 3D printed structures. The reviewer can see two significant aspects: the internal structures

of the 3D printing networks and the mechanical properties. After the degradation and washing away, how the porosity and crosslinking density can change? Consequently, how this would affect the mechanical strength and modulus of the 3D printing products? These are very important parameters for the eventual application of the 3D printing materials. However, they are apparently neglected. Moreover, how is the resolution and surface smoothness can be influenced? A more thorough study may be needed, regarding that numerous advanced volumetric and dual-wavelength 3D printing techniques have been developed with great merits already. For molecular synthesis, 13C NMR and HRMS were not provided.

We appreciate the reviewer's thoughtful questions regarding our degradable support strategy and its impact on the final mechanical properties, porosity, resolution, and surface smoothness of the printed parts, as well as their request for additional synthesis characterization. We have included the H-NMR (Fig S1) and 13C-NMR (Fig S2) for the synthesized crosslinker in the Supplementary Information section 1.1 and address their concerns below.

- **Effects on porosity and surface smoothness**

Our dual-wavelength resin formulation contains 80 wt% of epoxy monomers, which rely on UV initiated cationic polymerization, and 20 wt% of (meth)acrylate monomers that polymerize with visible light. Only the (meth)acrylated anhydride crosslinker is susceptible to base degradation, thus leaving the majority epoxy network (80 wt%) physically and chemically unaffected following base treatment. This was demonstrated in Fig S7, which shows that mass loss for 365 nm cured materials was equivalent to ~20 wt% after 3 hrs of degradation in 5M NaOH solution. We anticipate that the mass loss of the (meth)acrylated network accounts for 20 wt% porosity in the final epoxy parts.

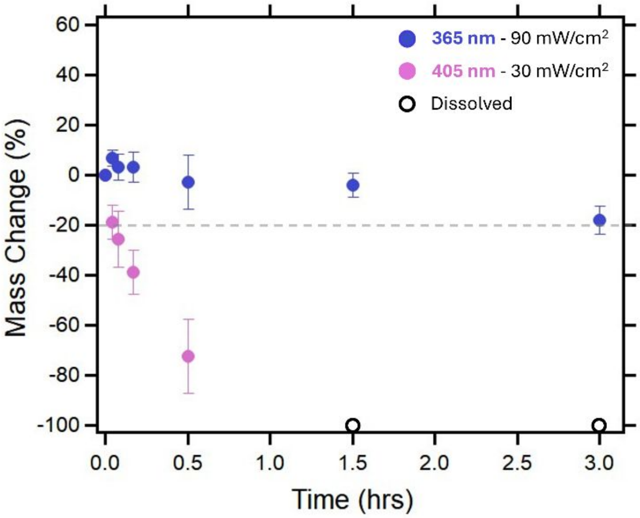

Figure S7. Mass loss data for 405 nm and 365 nm irradiated materials in 5M NaOH solution.

We obtained microscope images of the 365 nm cured parts before and after the degradation process (Fig S10). From the top view, the degraded parts appeared smoother and more sharply defined possibly due to the removal of unspecific cured regions formed by scattered light. In contrast, the side view at higher magnification shows layer-to-layer discontinuities which may arise from epoxy

conversion gradients along the z-axis. Regions that are less cured with 365 nm light are more susceptible to dissolution, which creates a stepped appearance between cured layers and voids in between. At higher magnification on the side view, we extracted surface roughness profiles both across multiple layers (Fig S11B, S11D) and along a single layer (Fig S11A, S11C) after post-processing and base degradation. The surface roughness profile along a single layer showed a relatively homogenous layer before (Fig S11A) and post-degradation (Fig S11C) with a variability of ~12-14 µm. In contrast, the surface roughness profile across multiple layers showed higher levels of variability after base degradation of ~40 µm (Fig S11D). Future work to address the discontinuity artifacts could include optimizing print parameters by using smaller layer thicknesses to improve epoxy conversion and uniformity between layers.

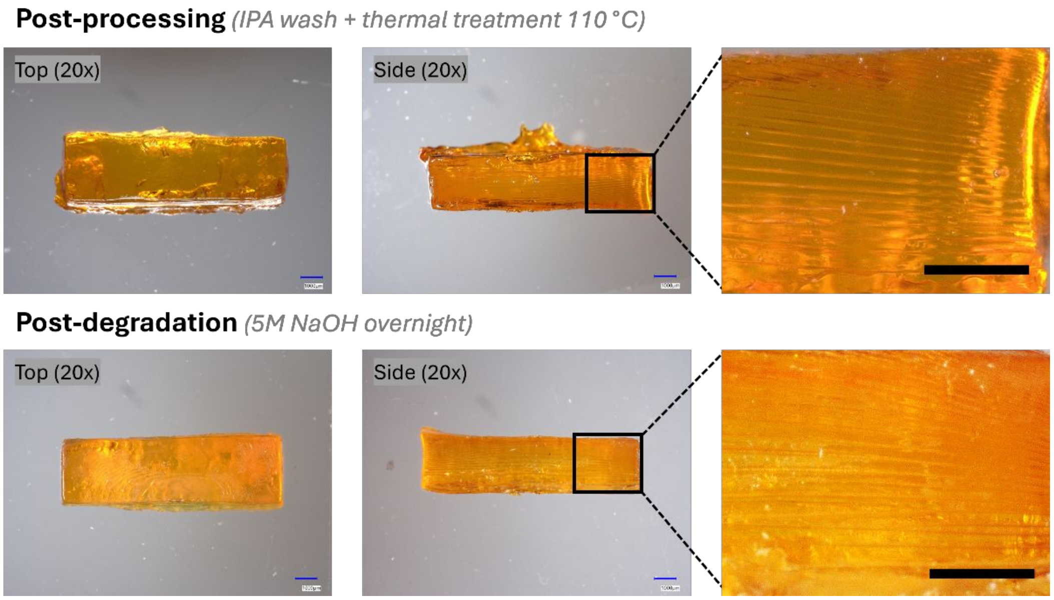

Figure S10. Microscope images of 365 nm-cured parts after post-processing (top row) and post-degradation (bottom row). Top views (20x) reveal smoother, sharper outlines after (meth)acrylate network removal. Side views (20, 80x) show layer-to-layer discontinuities post-degradation. Scale bars are 1 mm.

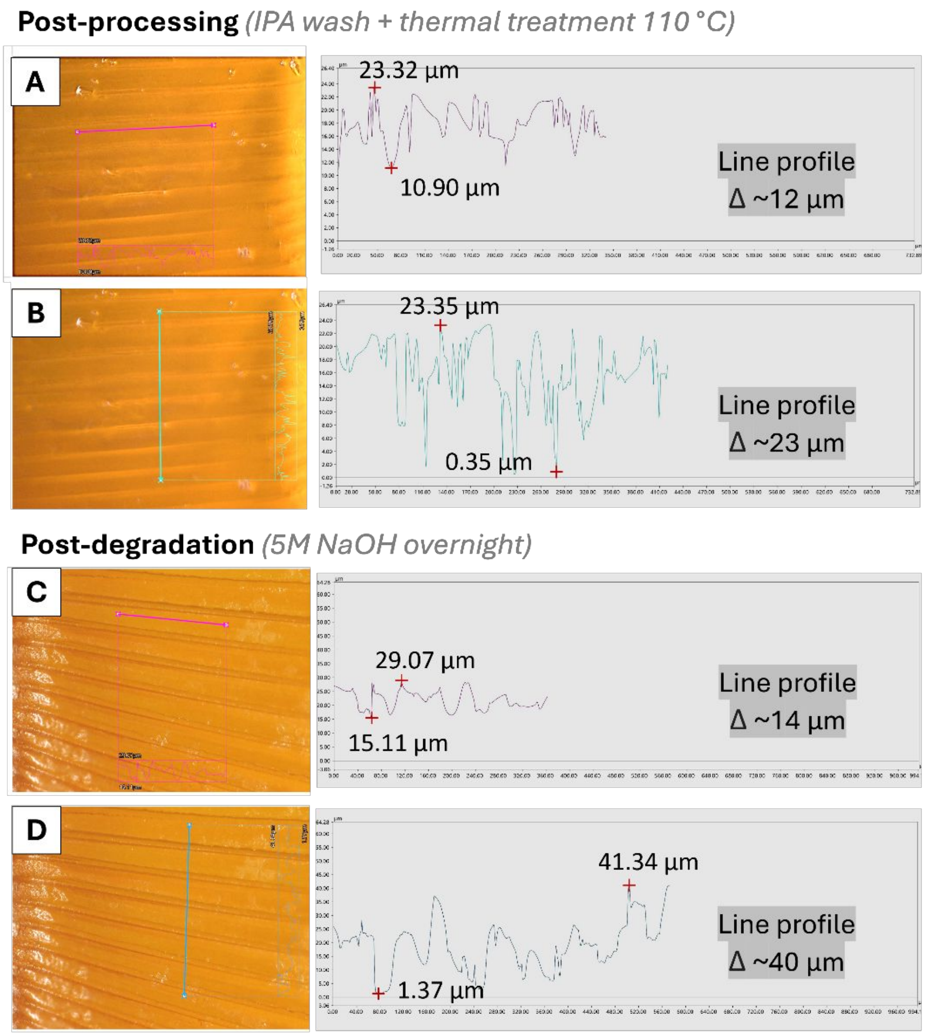

Figure S11. Surface roughness profiles both across multiple layers and along a single layer after post-processing steps (A, B) and post-degradation with 5M NaOH (C, D). The surface roughness profile across multiple layers (B, D) showed higher levels of variability post-degradation, while the surface roughness profile along a single layer (A, C) showed similar variability before and after degradation.

Effects on crosslinking density, mechanical strength, and modulus

Although some microporosity forms in the UV-cured parts post-degradation, the overall mechanical strength and stiffness of the final prints remain governed by the robust epoxy network. Dynamic mechanical analysis (DMA) of the parts before degradation shows a rubbery modulus of 2.69×10^7 Pa at 200 °C, corresponding to a crosslink density of approximately 2278 mol/m³. After base-mediated degradation of the (meth)acrylate network, the modulus slightly decreases to 1.74×10^7 Pa, reflecting a crosslink density of 1474 mol/m³. A modest decrease in glass transition temperature is also observed, from 152.1 °C to 139.9 °C, further indicating partial loss of the degradable network. Despite a ~35% reduction in the crosslink density, the bulk thermomechanical performance is largely preserved, indicating that the permanent epoxy network continues to

dominate the mechanical properties of the final parts even after removal of the degradable network. Due to the limited projection area of the DWNI printer, samples with suitable length for DMA testing were prepared by casting the resin into silicone molds. The resin was cured under 365 nm LED light with exposure energy matched to the printing process (6300 mJ/cm^2; 50 µm layer) to ensure comparable network structure.

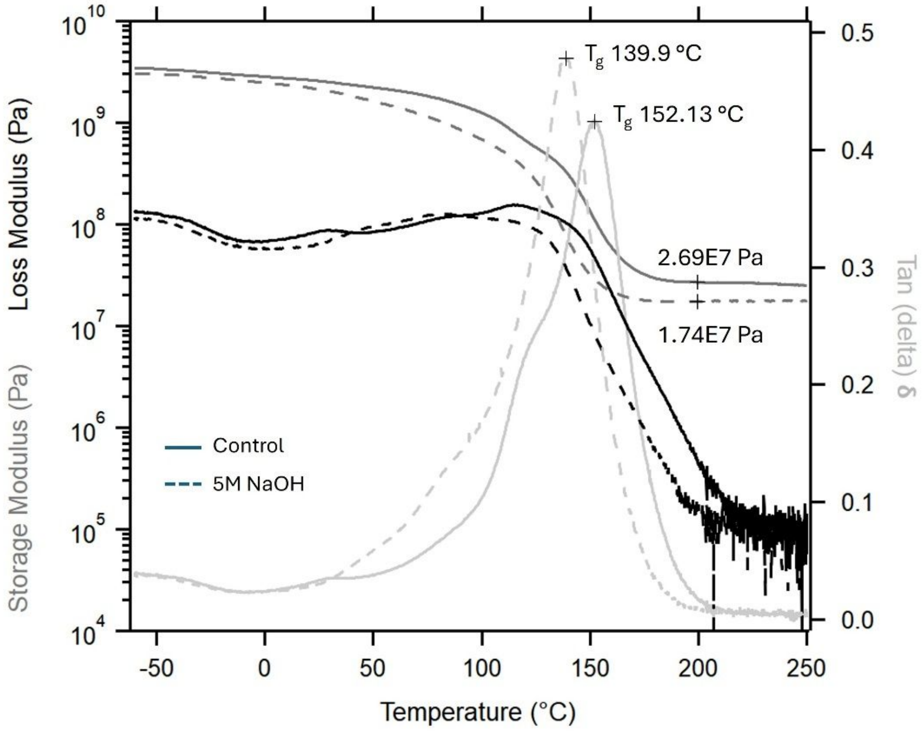

Figure S12. Dynamic mechanical analysis (DMA) curves of printed parts before (solid line) and after (dotted line) degradation showing loss modulus, storage modulus and tan (delta).

- **Effects on resolution**

The selective degradation of the (meth)acrylate network by the base treatment enhances resolution and dimensional accuracy by eliminating unintended crosslinking in areas affected by scattered light exposure (Fig S13, top and middle). In addition, exposure to both visible and UV light appears to improve feature resolution (smallest feature: 210 µm; Fig S13, bottom) and print fidelity compared to UV-only exposure (smallest feature: 260 µm; Fig S13, middle). This improvement may arise from the formation of the degradable (meth)acrylate network that temporarily constrains the epoxy network during curing, allowing for more precise shape retention and higher dimensional accuracy.

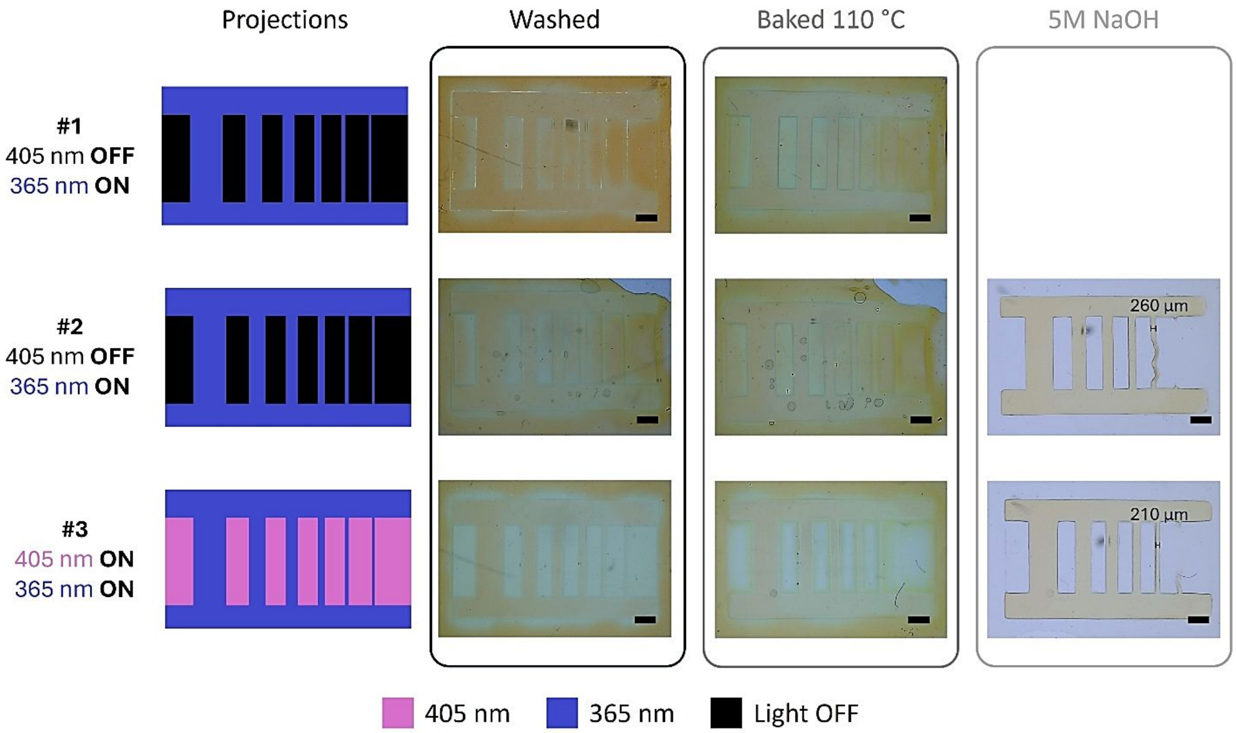

Figure S13. Resolution tests showing UV cured patterns under varying conditions, with (1) 405 nm OFF and 365 nm ON without post-degradation, (2) 405 nm OFF and 365 nm ON with post-degradation, and (3) 405 nm ON and 365 nm ON with post-degradation.
